# ACLY regulates autolysosome acidification through tubulin acetylation‐mediated assembly of V‐ATPase subunits in Alzheimer's disease model mice

**DOI:** 10.1002/alz.70919

**Published:** 2025-11-19

**Authors:** Anlan Lin, Xiaoman Dai, Jianmin Chen, Tianqing Han, Qiang Du, Minxia Wu, Jinbo Cheng, Wanjin Chen, Qinyong Ye, Xiaochun Chen, Jing Zhang

**Affiliations:** ^1^ Department of Neurology Fujian Medical University Union Hospital Fujian Key Laboratory of Molecular Neurology and Institute of Neuroscience Fujian Medical University Fuzhou China; ^2^ The School of Basic Medical Sciences Public Technology Service Center Fujian Medical University Fuzhou China; ^3^ Department of Hepatobiliary Surgery and Fujian Institute of Hepatobiliary Surgery Fujian Medical University Union Hospital Fuzhou China; ^4^ Beijing Institute of Basic Medical Sciences Beijing China; ^5^ Department of Neurology and Institute of Neurology The First Affiliated Hospital of Fujian Medical University Fuzhou China

**Keywords:** Alzheimer's disease, ATP citrate lyase, lysosomal acidification, vacuolar adenosine triphosphatase

## Abstract

**INTRODUCTION:**

Faulty autolysosome acidification leads to dystrophic neurites—an early event propelling Alzheimer's disease (AD) progression—yet the underlying mechanism remains elusive.

**METHODS:**

To elucidate the physiological functions of neuronal ATP citrate lyase (ACLY) expression, its impact on amyloid beta (Aβ) pathology, and molecular mechanisms, we used intracerebroventricular ACLY inhibitor administration, adeno‐associated virus–mediated ACLY modulation in the dorsal hippocampus, and N2a‐swAPP695 cell line.

**RESULTS:**

Inhibition or knockdown ACLY reduced microtubule stability and impaired cognition in wild‐type mice. Neuronal ACLY decreased in both AD patients and mice. ACLY knockdown in young 5×FAD mice exacerbated dystrophic neurites, aggravated Aβ deposition, and obstructed autophagic‐lysosomal flux. Conversely, enhancing ACLY improved cognition in advanced 5×FAD mice. Mechanistically, ACLY regulates lysosomal vacuolar adenosine triphosphatase assembly and acidification through α‐tubulin acetylation.

**DISCUSSION:**

Neuronal ACLY maintains microtubule stability and cognition, while critically regulating lysosomal acidification‐mediated amyloid pathology. These findings reveal novel mechanisms linking lysosomal dysfunction to AD, offering therapeutic insights.

**Highlights:**

- ATP citrate lyase (ACLY) as highly expressed in the processes of hippocampal neurons is essential for maintaining learning and memory through tubulin acetylation‐mediated microtubule stability.
- ACLY deficiency obstructed autophagic–lysosomal flux, aggravated amyloid beta deposition, and exacerbated dystrophic neurites in the early stages of Alzheimer's disease (AD).
- Enhanced neuronal ACLY promoted synaptic plasticity and alleviates cognitive impairment in AD mice with advanced neuropathology.
- ACLY regulates lysosomal vacuolar adenosine triphosphatase subunit assembly and lysosomal acidification via α‐tubulin acetylation in the AD brain.

## 1 BACKGROUND

Alzheimer's disease (AD) is neuropathologically characterized by two main types of lesions: intracellular tau aggregates, known as neurofibrillary tangles, and neuritic plaques consisting of extracellular amyloid beta (Aβ), dystrophic neurites, and various other proteins.^1^, ^2^ Importantly, dystrophic neurites (DNs) originate from axons,^3^, ^4^ and abnormally accumulate enlarged autophagic vacuoles (AVs) containing incompletely digested autophagy substrates, which block action potential conduction and disrupt neural network function.^5^ ^−^ ^8^ Despite growing evidence suggesting that presynaptic DNs are sites of lysosomes with deficient luminal proteases and impaired acidification, as well as elevated beta‐secretase 1 and increased Aβ generation in AD,^9^, ^10^ the mechanisms underlying lysosomal system dysregulation remain unclarified.

Luminal acidification of lysosomes to a pH of 4.5 to 5.0 is essential for their function.^11^ This acidification is primarily maintained by the vacuolar (H⁺)‐adenosine triphosphatase (V‐ATPase), a multimeric enzyme complex that pumps protons from the cytosol into the lysosomal lumen. The intact V‐ATPase complex consists of two sectors: the membrane‐associated V0 sector and the cytosolic V1 sector.^12^ V‐ATPase activity is regulated by various mechanisms that modulate subunit expression, assembly, trafficking, and signaling. Among these, the regulation of the assembly between the V0 and V1 sectors is essential for forming a functional V‐ATPase channel.^13^ The assembly of V‐ATPase is a reversible process dynamically regulated by metabolic signals, such as glucose starvation.^14^ The C subunit of the V1 domain of V‐ATPase directly interacts with microtubules, and the dissociation of the V1 from the V0 domain induced by glucose starvation is influenced by microtubule depolymerization agents.^15^, ^16^ This implies that microtubule stability, in addition to being involved in autophagosome transport and the fusion of autophagic/endocytic vesicles with lysosomes,^17^, ^18^ may play a critical role in the acidification of lysosomes. Previous research has demonstrated early deficits in lysosomal V‐ATPase activity and the accumulation of Aβ selectively within poorly acidified autolysosomes (ALs) well before extracellular Aβ deposition.^19^, ^20^ To date, the mechanisms underlying deficiencies in lysosomal V‐ATPase activity in AD neurons have rarely been investigated.

Abnormal glucose and lipid metabolism are present in the brains of individuals with AD.^21^, ^22^ However, the relationship between these metabolic abnormalities and Aβ deposition remains unclear. Acetyl‐CoA is not only a key molecule in metabolic pathways but also serves as a donor for acetylation modifications of histone, cytoskeletal proteins, and messenger protein.^23^ Three principal enzymes are crucial for directly maintaining acetyl‐CoA levels: ATP‐citrate lyase (ACLY), acyl‐CoA synthetase short‐chain family member 2 (ACSS2), and the pyruvate dehydrogenase complex (PDC). Previous studies have reported that ACSS2‐dependent histone acetylation regulates hippocampal memory.^24^, ^25^ However, the physiological and pathological roles of ACLY, which catalyzes the direct conversion of mitochondrial tricarboxylic acid cycle–derived citric acid to acetyl‐CoA, in the central nervous system have not yet been addressed.

In this study, inhibition of ACLY enzyme activity or hippocampal neuronal ACLY knockdown led to impaired learning and memory in wild‐type (WT) mice. This impairment was associated with decreased tubulin acetylation and reduced microtubule stability. Additionally, we observed reduced expression and enzyme activity of neuronal ACLY in both AD brains and 5×FAD mice. Notably, increased levels of neuronal ACLY stabilize microtubules, enhance autophagic–lysosomal activity, and significantly reduce DNs and Aβ deposition, thereby improving learning and memory in 5×FAD mice. Further, in the APP695‐N2a cell line and primary cultured neuron, we investigated the role of ACLY in regulating the assembly of lysosomal V‐ATPase subunits through acetylated tubulin, which affects lysosomal function and the pathological process of Aβ. The results revealed the physiological role of the key metabolic enzyme‐ACLY in the brain and its relationship with the characteristic pathological features of classical neurodegeneration. Our findings, together with other emerging data,^26^, ^27^ point to a unifying pathogenic mechanism underlying primary lysosomal dysfunction in AD.

## 2 MATERIALS AND METHODS

### 2.1 Animal and human *post mortem* tissue

The 5×FAD mouse model, which co‐expresses five familial AD mutations in the human amyloid precursor protein (K670N/M671L [Swedish], I716V [Florida], and V717I [London]) and human presenilin 1 (M146L, L286V) under the control of the murine Thy‐1 promoter, was obtained from the Jackson Laboratory (stock no. 034848 JAX). Genotyping was performed by polymerase chain reaction (PCR) analysis of tail DNA, following methods previously described.^28^ All AD mice and their littermate controls used in this study were male. The animals were housed in groups of up to five per cage and maintained under a standard 12 hour light/dark cycle at a temperature of 22 ± 1°C. All animal procedures were approved by the institutional animal care and use committee of Fujian Medical University and were conducted in accordance with international ethical guidelines for animal research.

*Post mortem* brain tissue from individuals with AD and normal controls were obtained from the National Human Brain Bank for Development and Function at the Chinese Academy of Medical Sciences and Peking Union Medical College in Beijing, China. Detailed demographic and clinical data are provided in Table S1 in supporting information. The ethics board of the Institute of Basic Medical Sciences, Chinese Academy of Medical Sciences, and Peking Union Medical College (approval no. 009‐2014) approved the use of these tissues.

**RESEARCH IN CONTEXT**

1. **Systematic review**: Neuritic plaques consisting of extracellular amyloid beta (Aβ), dystrophic neurites, and various other proteins are a central neuropathology of Alzheimer's disease (AD). Despite growing evidence suggesting that presynaptic dystrophic neurites are sites of lysosomes with deficient luminal proteases and impaired acidification, as well as elevated beta‐secretase 1 and increased Aβ generation in AD, the mechanisms underlying lysosomal system dysregulation remain unclarified.
2. **Interpretation**: Here, we identified a critical role for neuronal ATP citrate lyase (ACLY) in regulating lysosomal vacuolar‐adenosine triphosphatase assembly and acidification that triggers neurodegeneration in AD. Our findings position ACLY as a master regulator of AD‐linked cytoskeletal–lysosomal crosstalk. Targeting ACLY‐mediated acetylation pathways offers a dual‐pronged therapeutic strategy: stabilizing microtubules to preserve neuronal connectivity while restoring lysosomal acidification to clear Aβ aggregates.
3. **Future directions**: Furthermore, given the central role of ACLY in metabolism and messenger molecules, implications for future studies to define metabolic dysregulation in AD pathogenesis could represent paradigm‐shifting views of how metabolism can potentially influence degenerative events in the brain.

### 2.2 Intraventricular catheter drug administration

Mice were anesthetized with isoflurane and positioned in a stereotaxic frame. Anesthesia was maintained via a nose cone connected to a gas delivery system. Respiratory rate was monitored throughout the procedure to ensure anesthetic depth and animal well‐being. A longitudinal midline incision was made in the scalp to expose the skull and identify the bregma. The periosteum was gently removed, and the skull surface was cleaned and dried. The stereotaxic needle was positioned at the bregma to set the zero point. A burr hole was drilled at the following coordinates relative to bregma: AP ‐0.6 mm, ML ±1.1 mm. The hole was carefully expanded with a fine surgical drill to precisely fit the cannula. The guide cannula was slowly lowered to a DV coordinate of ‐1.7 mm from the skull surface. It was first secured to the skull with cyanoacrylate adhesive applied to its base, and then permanently fixed by building a head cap using dental cement. The underlying skin was gently separated from the dental cement to prevent post‐operative tension. After the cement had hardened, the scalp incision was sutured closed, and a dummy cannula was inserted.

Following surgery, mice were single‐housed in a temperature‐controlled recovery chamber until fully ambulatory and received postoperative analgesia for up to 48 hours. After a 7‐day recovery period, mice received daily intracerebroventricular (ICV) infusions of the ACLY inhibitor BMS‐303141 (0.84 mg/kg) for 7 consecutive days. Cognitive behavioral testing began on day 8 following the final drug administration. Immediately upon completion of the tests, mice were euthanized, and brain tissues were collected for subsequent biochemical analysis. The cannula was inserted into the prepared hole, and a small amount of adhesive was applied to the base of the cannula holder to secure it to the skull. The area around the cannula was sealed with dental cement. The skin at the rear of the incision was quickly separated from the cement to prevent any tension on the cannula from neck movements. Once the cement hardened, the skin at the incision site was sutured and a cap was placed on the cannula. After completing the procedure, the mouse was removed from the stereotaxic frame and placed in a warming chamber. The mice regained consciousness and resumed normal activity within a few minutes. After a 7 day postoperative recovery, conscious animals underwent daily intracerebroventricular (ICV) administration of the ACLY inhibitor BMS‐303141 (0.84 mg/kg) for 7 consecutive days. Cognitive behavioral assessments were initiated on day 8 post‐treatment, with brain tissue collection immediately after testing for subsequent biochemical analyses.

### 2.3 Stereotaxic virus injection

Both 5×FAD and WT mice were anesthetized with isoflurane and secured in a stereotactic frame (Stoelting, USA). A total of 2 × 10^12^ viral genomes (vg) per site in a small volume were bilaterally injected into the CA1 region of the dorsal hippocampus (dHip; AP, −1.9 mm from bregma; DV, −1.15 mm from skull surface; ML, ±1.45 mm from midline) at a rate of 50 nL/minute using a pulled glass capillary and pressure microinjector. The capillary was left in place for 5 minutes after the injection to ensure proper diffusion before removal. The viral vectors used included: AAV2/9‐hSyn‐*ACLY*‐3×FLAG‐tWPA (5×FAD‐oe*ACLY*), AAV2/9‐hSyn‐MCS‐3×FLAG‐tWPA (control), AAV2/9‐hSyn‐EGFP‐3×flag‐miR30shRNA (*ACLY*)‐WPRE (5×FAD‐sh*ACLY*), and AAV2/9‐hSyn‐EGFP‐3×flag miR30shRNA(scrambled)‐WPRE (control), all designed and synthesized by OBiO Technology Corp., Ltd.

### 2.4 Behavioral tests

#### 2.4.1 Y‐maze test

Mice were held in the palm of the hand and gently stroked for 1 to 2 minutes daily over a period of 7 consecutive days. This process helped the animal fully acclimate to the experimenter and the surrounding environment, thereby reducing any signs of fear. After this acclimation period, mice were positioned in the center of a Y‐maze with their heads randomly oriented toward one of the arms, and a 5 minute timer was initiated. During this time, the path taken by each mouse was recorded. Finally, arm entry patterns and rotation indices of the mice within the Y‐maze were analyzed using EthoVision XT16 software.

#### 2.4.2 Morris water maze test

The Morris water maze (MWM) test was used to evaluate spatial learning and memory in mice, following a slightly modified version of an established protocol.^29^ The test took place in a dark, circular pool measuring 1.2 m in diameter and 0.5 m in height, filled with opacified water to a depth of 35 cm. The water level was set 1.5 cm above a transparent platform, 7 cm in diameter, located at the center of the southeast quadrant. The water temperature was kept at 22 ± 2°C. Visual cues, including various objects with distinct shapes and colors, were affixed to the walls of each quadrant.

During the training phase, each mouse underwent four trials per day for 5 consecutive days. The starting point for each trial varied according to the semi‐random sequence recommended by Vorhees and Williams^99^, with the mice always placed facing the pool wall. Mice were given 60 seconds to find the hidden platform; if they failed, they were gently guided to the platform and allowed to remain there for 20 seconds. The latency time was calculated as the average of the four trials. Twenty‐four hours after the final training session, a memory retention test was conducted with the platform removed. Each mouse was given 60 seconds to explore the pool, and their swimming behavior was recorded and analyzed using EthoVision video tracking software (Noldus).

### 2.5 Electrophysiology

Electrophysiology was performed as described in the references.^30^ 5×FAD and WT mice were anesthetized with isoflurane, decapitated, and their brains were removed and placed on ice oxygenated at < 4°C. The brains were then perfused with artificial cerebrospinal fluid (ACSF). Tissue blocks containing the hippocampus were fixed in a vibrating microtome's slicing slot using 502 glue, and ice‐cold ACSF was added. The hippocampal regions were sectioned into 300 µm thick transverse slices using the vibrating microtome. The brain slices were incubated in continuously oxygenated ACSF (95% O_2_ and 5% CO_2_) for at least 1.5 hours before recording.

After incubation, the slices were placed into an interfacial recording chamber supported by a nylon mesh and continuously perfused with ACSF at a flow rate of 1 to 2 mL/minute. The ACSF was maintained at a temperature of 30 ± 1°C, with a steady supply of 95% O_2_ and 5% CO_2_. A bipolar stainless steel stimulating electrode was positioned on the Schaffer collateral pathway within the hippocampal CA1 region, while a recording microelectrode was inserted ≈ 200 µm below the slice surface in the same region. The microelectrode was filled with 3 mol/L NaCl and had an impedance ranging from 2 to 5 MΩ. Field potentials were amplified using a microelectrode amplifier, and the output signals were recorded, analyzed, and processed with the Axon Digidata 1550B data acquisition system. Stimulation consisted of a square wave, 0.1 ms in width, at a frequency of 0.1 Hz. The stimulus intensity was adjusted to elicit a synaptic response equal to half of the maximum synaptic potential. Long‐term potentiation (LTP) was induced by delivering two trains of high‐frequency stimuli at 100 Hz, each lasting 1 second, with a 10 second interval between trains. Both 5×FAD and WT mice were used to assess differences in synaptic plasticity.For recording, a single slice was transferred to an interfacial chamber, continuously perfused with ACSF (1‐2 mL/min) maintained at 30 ± 1°C and saturated with 95% O_2_ / 5% CO_2_. A bipolar stainless‐steel stimulating electrode was positioned on the Schaffer collateral pathway. A glass microelectrode (2‐5 MΩ impedance) filled with 3 M NaCl was placed approximately 200 µm below the slice surface in the CA1 stratum radiatum for recording. Field excitatory postsynaptic potentials (fEPSPs) were amplified and acquired using an Axon Digidata 1550B system. Stimuli (0.1 ms square waves at 0.1 Hz) were delivered at an intensity that elicited an fEPSP slope 50% of the maximum. Long‐term potentiation (LTP) was induced by applying two 1‐second, 100 Hz trains with a 10‐second inter‐train interval. Recordings were compared between slices from 5×FAD and WT mice.

### 2.6 Acetyl‐CoA enzyme‐linked immunosorbent assay

Acetyl‐CoA levels in the hippocampus or dHip were measured using a mouse Ac‐CoA enzyme‐linked immunosorbent assay (ELISA) Kit (Shanghai Yiyan Biotech Co., Ltd, EY12009‐M) following the manufacturer's instructions.

### 2.7 ACLY enzyme activity test

ACLY activity was measured using the malate dehydrogenase coupling method.^31^ Tissue samples (≈ 25 mg) were combined with a reaction mixture containing ATP (200 mmol/L Tris‐HCl, pH 8.7; 20 mmol/L MgCl_2_; 20 mmol/L potassium citrate; 1 mmol/L DTT; 0.2 mmol/L NADH; and 1 U/mL MDH) and incubated with 0.5 mmol/L CoA. Enzyme activity was assessed at 3 minute intervals over a 30 minute period using a NanoDrop 2000c spectrophotometer (Thermo Fisher Scientific). Specific ACLY activity was calculated as the change in absorbance with ATP minus the change in absorbance without ATP, normalized to protein concentration.

### 2.8 Quantification of Aβ_42_ levels by ELISA

Hippocampal samples used for ELISA were prepared as previously described with some modifications.^32^ The separated hippocampal tissue was added to 300 µL of pre‐cooled TBST lysis buffer (TBS pH 7.4, 1% Triton X‐100, 50 mM NaF, 2 mM sodium orthovanadate, 10 mM sodium pyrophosphate, 1% protease inhibitor mixture). The tissue was homogenized using an ultrasonic crusher (parameter settings: frequency 100 W, working time 1 second, interval time 1 second, 20 cycles). After homogenization, the tissue samples were placed on ice for 30 minutes for lysis. The samples were then centrifuged in a refrigerated ultracentrifuge (4°C, 16,000 × g, 25 minutes). The supernatant was transferred to an EP tube, labeled as TBST‐dissolved Aβ_42_ (TBST‐Aβ_42_), and stored at −80°C. The precipitate was subjected to another round of centrifugation in the refrigerated ultracentrifuge (4°C, 16,000 × g, 5 minutes), and the supernatant was discarded. The precipitate was then treated with 400 µL of 5 M guanidine hydrochloride solution. Aβ_42_ levels in the hippocampus or dHip were measured using a Human Aβ_42_ ELISA Kit (Invitrogen, KHB3441) according to the manufacturer's instructions.

### 2.9 Aβ staining

After washing the brain sections three times with TBS, the sections were incubated in 50% alcohol‐diluted Thioflavine S (Merck T1982‐25G) or Methoxy‐X04 (MCE HY‐103240) at room temperature, avoiding light, for 8 minutes. Then, the sections were washed with 50% alcohol, followed by three washes with TBS. Finally, an anti‐fading agent was applied and the sections were mounted.

### 2.10 Cathepsin L activity

MagicRed (ICT‐941) is a fluorescence probe specifically designed to detect protease L activity. The experiment began with the digestion and passage of cells, which were then transferred to a suitable dish for confocal microscopy imaging. Once the cell density reached ≈ 50% (an optimal cell density yields more reliable results), the culture medium was removed, and the pre‐warmed MagicRed working solution at 37°C was added. According to the product instructions, the MagicRed dye was diluted to a 1× concentration with culture medium and incubated with the cells for 20 to 30 minutes. After incubation, the cells were removed and washed two to three times with 0.01 M phosphate‐buffered saline (PBS) to remove any unbound dye. Finally, confocal microscopy imaging was performed to observe the activity of protease L.

### 2.11 Lysosome pH measurement

Lysosensor is a pH‐dependent fluorescent probe with dual‐excitation and dual‐emission properties. First, the cells were digested, passaged, and transferred to a confocal dish to allow them to grow. When the cell density reached ≈ 70% to 80% (with better results at higher density), the culture medium was removed, and an appropriate amount of the probe‐containing working solution, preheated to 37°C, was added. A mixture of 1 mL medium and 1.5 µL dye was preheated and incubated for 3 minutes. The cells were then washed two to three times with 0.01 M PBS buffer, and confocal imaging was performed.

### 2.12 Electron microscope sample preparation

The sample was removed and placed in a Petri dish. After the addition of glutaraldehyde, it was cut into trapezoidal pieces with a blade. Tissue pieces smaller than 1 mm^3^ were transferred to 2 mL EP tubes, and ≈ 1 mL of 2.5% glutaraldehyde was added for fixation for at least 2 hours. The sample was then rinsed seven to eight times with 1 mL of 0.01 M PBS buffer for 15 minutes per rinse. Subsequently, 1 mL of tetroxide fixing solution was added, and the sample was fixed overnight. The EP tubes were rinsed again five to six times with 1 mL of 0.01 M PBS buffer for 15 minutes each time.

The sample was dehydrated in a graded ethanol and acetone series: 30% ethanol for 15 minutes, 50% ethanol for 15 minutes, 70% ethanol for 15 minutes, 90% ethanol for 15 minutes, 100% ethanol for 20 minutes, and 100% acetone for 20 minutes. It was then infiltrated with different proportions of acetone and embedding agent: 1:1 for 1 hour, 1:3 for 1 hour, and pure embedding agent for 2 hours. The sample were transferred to a new test tube using a toothpick and placed in a polymerization box for 4 hours. After removal, the sample was placed into a mold.

The sample was roughly trimmed with a blade and then finely trimmed on an ultra‐thin microtome (Leica UC6) at a speed of 1 mm/second to achieve a thickness of 50 to 70 nm. The sections were collected on a copper grid and mounted on a silica gel plate. After double staining with lead and uranium, the sections were observed using an electron microscope (Hitachi High‐Tech HT7700, voltage 220 V, current 10 µA).

### 2.13 Western blotting

Hippocampal brain tissues were sonicated in chilled radioimmunoprecipitation assay buffer (Abcam, #ab156034) supplemented with protease inhibitors (Millipore, #539131), phenylmethanesulfonyl fluoride (CST, #8553), phosphatase inhibitors (MCE, #HY‐K0022), and deacetylase inhibitors (5 mM nicotinamide, 1 µM trichostatin A). After sonication, the lysates were subjected to centrifugation at 16,000 × g for 25 minutes at 4°C. The resulting supernatants were collected. Protein concentrations were quantified via a bicinchoninic acid assay (Beyotime, #P0009) and normalized accordingly.

The samples were subjected to heating at 100°C for 10 minutes. Proteins, in equal quantities, were then resolved by sodium dodecyl sulfate polyacrylamide gel electrophoresis and transferred onto polyvinylidene fluoride membranes. Blocking was performed using 5% bovine serum albumin (BSA) in TBST at room temperature for 1 hour, followed by an overnight incubation at 4°C with primary antibodies diluted in 5% BSA/TBST. Afterward, the membranes were washed three times with TBST and incubated with horseradish peroxidase‐conjugated secondary antibodies at room temperature for 1 hour. Immunoblot signals were developed using an enhanced chemiluminescence substrate and quantified using National Institutes of Health ImageJ software. The specific antibodies used are detailed in Table S2 in supporting information.

#### 2.13.1 Membrane plasma separation

A Thermo Scientific Cell Membrane Protein Extraction and Isolation Kit (Thermo, YD371740), which includes cell wash buffer, solubilization buffer, permeabilization buffer, protease inhibitor (PIC), phosphatase inhibitor (PPIC), cell scrapers, 1.5 mL EP tubes, and 2 mL/15 mL centrifuge tubes was prepared. Twenty to 40 mg of hippocampal tissue was placed in a 15 mL centrifuge tube, 4 mL of cell wash buffer was added, vortexed, and then discarded. Using scissors, the tissue was cut into small pieces and thoroughly ground. One mL of permeabilization buffer was added to the tissue, grinding continued, and then it was pipetted until a homogeneous suspension formed An additional 1 mL of permeabilization buffer was added and then incubated at 4°C for 10 minutes with continuous mixing, vortexing every 5 minutes. The permeabilized tissue was centrifuged using a refrigerated ultracentrifuge at 4°C, 16,000 × g, for 15 minutes. The supernatant, which contains cytoplasmic proteins, was gently collected and transferred to a fresh 1.5 mL EP tube. The remaining pellet was resuspended in 1 mL of solubilization buffer to create a uniform suspension, then incubated at 4°C for 30 minutes with continuous agitation. After incubation, the sample was centrifuged again at 16,000 × g and 4°C for 15 minutes. The supernatant, which now contains the soluble membrane proteins and membrane‐associated proteins, was transferred into a new 1.5 mL EP tube and stored at –80°C.

### 2.14 Immunofluorescence assays

Coronal sections of the hippocampal region were sliced using a freezing microtome. After cutting, the sections were washed three times in and then blocked for 1 hour at room temperature in a solution containing 0.3% Triton X‐100, 1% BSA, and 10% normal donkey serum. Next, the sections were incubated overnight at 4°C with primary antibodies diluted in TBS containing 0.3% Triton X‐100, 1% BSA, and 2.5% normal donkey serum. After this, the sections were washed three times with TBST and incubated for 1 hour at room temperature with secondary antibodies. After washing, the sections were counterstained with 4′,6‐diamidino‐2‐phenylindole. Confocal images were acquired using a Zeiss microscope. Antibody details can be found in Table S2.

### 2.15 Cell culture

#### 2.15.1 Primary cultured neurons

Primary neurons were isolated from the brains of C57BL/6J mice embryos at day 18 (E18). Hippocampal tissue was dissected under a microscope, and the meninges were removed while submerged in Dulbecco's modified Eagle medium (DMEM)/F12 medium (HyClone, SH30023.01B). The tissue was then digested in a solution containing 200 µL of papain suspension (Worthington, LS003126), DNase (Worthington, LS002139), and cysteine (Sigma, C1276), all mixed into 12 mL DMEM/F12. This digestion process was carried out at 37°C with 5% CO_2_ for 50 minutes, with gentle shaking every 10 minutes. Post‐digestion, the tissue was allowed to settle, and the supernatant was removed. The remaining tissue was resuspended in 10 mL DMEM/F12 and mechanically dissociated by pipetting. After a 2 minute settling period, 2.5 mL of supernatant was collected and transferred into a fresh tube. This procedure was repeated three times using fresh DMEM/F12 medium. The combined supernatants were then passed through a 70 µm sieve (Falcon, 352350) and spun down at 400g for 5 minutes. After discarding the supernatant, the cells were resuspended in Neurobasal medium supplemented with 1 mL B27 (Gibco, 17504‐044) and 0.5 mL of 100× glutamine (Gibco, 25030‐081) for subsequent culturing.

#### 2.15.2 Primary cultured astrocytes and microglia

One‐day‐old C57BL/6J mice were placed in sterile petri dishes kept on ice. After disinfection with alcohol, the mice were decapitated, and their brains were extracted. Under a dissection microscope, the cerebellum (Cb), brainstem, and olfactory bulb (OB) were removed, and the cerebral cortex and hippocampus were isolated, with the meninges stripped away. The isolated hippocampal tissue was transferred to a 15 mL centrifuge tube, and digestive fluid was added. The tissue was digested in a 37°C incubator for 20 minutes, with gentle agitation every 5 minutes. The tube was then centrifuged at 400 × g for 5 minutes, and the supernatant was removed and discarded. An equal volume of astrocyte culture medium was added, and the tissue was washed twice to halt the digestion process. The tissue was then resuspended in 2 mL of astrocyte culture medium and gently pipetted to disperse the cells. After allowing larger tissue pieces to settle, the supernatant was transferred to a clean centrifuge tube. The process was repeated until no visible tissue remained. The collected cells were counted, and the concentration was adjusted to 1–5 × 10⁵ cells/mL. The cells were then seeded into polylysine‐coated culture flasks and incubated at 37°C. The culture medium was changed after 24 hours and then every 3 days, with a complete medium change each time. After 14 days of in vitro culture, the mixed cell layer was shaken at 260 rpm for 1 to 2 minutes at room temperature to separate the microglia. The remaining cells in the original culture flasks were primary astrocytes. The supernatant cell suspension was collected and centrifuged at 500 × g for 8 minutes at 4°C, and the pellet was collected to obtain purified microglia.

#### 2.15.3 Neuro‐2a and N2a/APP 695 SWE

The Neuro‐2a (N2a) and N2a/APP 695 SWE cell lines were obtained from the following sources: the N2a cell line was provided by the Cell Bank, Type Culture Collection of the Chinese Academy of Sciences (CBTCCCAS), while the N2a/APP 695 SWE cell line was purchased from the HeYuan Company. Both cell lines were tested for mycoplasma contamination and authenticated by their respective suppliers. The cells were cultured in high‐glucose DMEM supplemented with 10% fetal bovine serum (FBS) and maintained at 37°C under 5% CO_2_.

#### 2.15.4 Tubulin‐KO N2a cells

The single guide RNAs (sgRNAs) were designed using the online tools CRISPOR (http://crispor.tefor.net/). We constructed the sgRNA‐Cas9‐CMV‐EGFP vector targeting the Tubulin locus. Transient transfection of N2a cells with vector or Tubulin‐Cas9 plasmids was performed using Lipofectamine 3000 (Thermo Fisher Scientific). The EGFP tag of the guide vector was used for single‐cell sorting. Single clones were subsequently obtained and validated through Sanger sequencing.

### 2.16 CCK‐8

The MCE Cell Counting Kit‐8, referred to as the CCK‐8 Kit (MCE HY‐K0301‐100T) was used for rapid and high‐sensitivity detection of cell proliferation and cytotoxicity without the use of radioactivity.

### 2.17 Real‐time PCR

Total RNA was extracted from the dHip or primary neuron cells using Trizol (Invitrogen, 15596018) according to the manufacturer's instructions. The extracted RNA was reverse‐transcribed into cDNA using the One‐Step Kit (Vazyme, R333‐01). Gene expression levels were quantified using SYBR Green I (Vazyme, Q712‐03) on a quantitative real‐time PCR thermocycler (Applied Biosystems, StepOnePlus). The primer used for the reactions can be found in Table S3 in supporting information. Relative gene expression levels were calculated using 2^ (‐Delta‐delta Ct), with multiple technical replicates and no‐template controls included in each experiment.

### 2.18 Data processing and statistical analysis

Data were represented as the mean ± standard error of the mean and analyzed using GraphPad Prism version 8.0. To assess the normality of the data, the Shapiro–Wilk test was applied, with most datasets showing a normal distribution. The homogeneity of variances was checked using the Bartlett test. For two independent, unpaired samples, if the data met the criteria of normality and equal variances, an unpaired *t* test was conducted; otherwise, the Mann−Whitney test was used. Comparing three or more independent groups, a one‐way analysis of variance (ANOVA) followed by a Tukey post hoc test was performed when both normality and homogeneity of variances were present. For datasets with unequal variances, Welch ANOVA followed by a Tamhane *T2* test was conducted. If the data deviated from normality, the Kruskal−Wallis test followed by a Dunn post hoc test was used. For multiple groups with two factors, two‐way ANOVA followed by either a Tukey or Bonferroni test was used if the data were normally distributed and had equal variances; otherwise, non‐parametric tests were used. A *P* value < 0.05 was considered statistically significant.

## 3 RESULTS

### 3.1 Decreased neuronal ACLY expression and enzyme activity in AD patients and AD model mice

**FIGURE 1:**
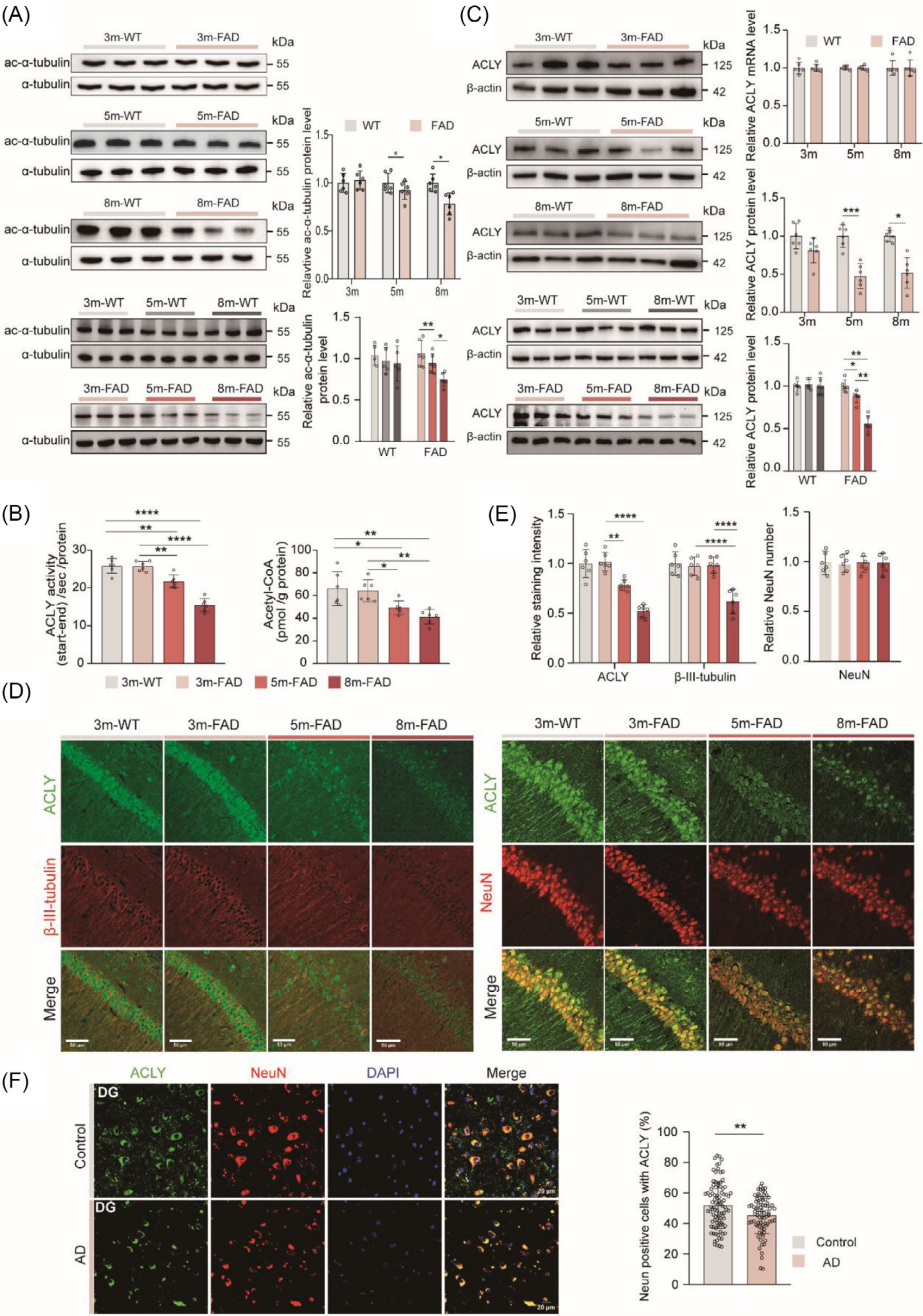
Decreased neuronal ACLY expression and enzyme activity in AD patients and 5×FAD mice. A, Representative immunoblots and quantitative analyses of ac‐α‐tubulin/α‐tubulin in the dHip; *n *= 6 mice per group. B, Detection of ACLY enzymatic activity (left) and quantification of acetyl‐CoA level (right) in the dHip; *n *= 6 mice per group. C, Representative immunoblots and quantitative analyses of ACLY in the dHip. Quantitative analyses of ACLY mRNA in the dHip; *n* = 6 mice per group. D−E, IF staining and quantitative analyses of ACLY (green), β‐III‐Tubulin (red), NeuN (red) in the hippocampal CA1 region; scale bar = 50 µm; *n *= 6 slices from three mice per group. F, IF staining and quantitative analyses of ACLY and NeuN in the hippocampal DG of the human with AD and non‐AD; scale bar = 20 µm. Data were expressed as mean ± standard error of the mean. ^*^ *P *< 0.05, ^**^ *P *< 0.01, ^***^ *P *< 0.001, ^****^ *P *< 0.0001. Two‐tailed unpaired Student *t* test was used in (A), (C), and (F). One‐way analysis of variance with a Tukey multiple comparisons test was used in (A), (B), (C), and (E). ACLY, ATP‐citrate lyase; AD, Alzheimer's disease; DG, dentate gyrus; dHip, dorsal hippocampus; IF, immunofluorescence; WT, wild type

Tubulin acetylation is a significant post‐translational modification that primarily occurs at the K40 site of the α‐tubulin. This modification plays a crucial role in regulating microtubule stability, cytoskeletal functions, and intracellular transport processes.^33^, ^34^ Abnormalities in tubulin acetylation are associated with neurodegenerative diseases, including AD, Parkinson's disease (PD), and Huntington's disease (HD).^35^ ^−^ ^40^ However, the regulatory mechanisms involved remain unclear. First, we assessed the level of acetylated tubulin (ac‐tubulin) relative to total tubulin in the dHip of 5×FAD mice, a well‐established animal model of AD. Compared to littermate controls, 5×FAD mice exhibited a reduction in ac‐tubulin levels in the dHip at 5 months of age, with a progressive and age‐dependent decline (Figure 1A).[Fig alz70919-fig-0002] Tubulin acetylation is known to be regulated by acyltransferases, such as N‐acetyltransferase 3 (NAT3) and alpha‐tubulin acetyltransferase 1 (ATAT1), as well as deacetylases, including N‐deacetylase and N‐sulfotransferase 3 (NDST3) and histone deacetylase 6 (HDAC6).^41^, ^42^ Interestingly, no significant differences were observed in the mRNA levels of these enzymes between 5×FAD mice and their littermate controls at any age (Figure S1A in supporting information). Previous studies have demonstrated that acetyl‐CoA, derived from ACLY, serves as a critical substrate for tubulin acetylation in vitro.^43^ Notably, in the dHip of 5×FAD mice, both acetyl‐CoA levels and ACLY enzyme activity decreased in parallel with the reduction in ac‐tubulin (Figure 1B). Correspondingly, ACLY protein levels in 5×FAD mice declined in an age‐dependent manner, with a significant reduction evident at 5 months of age, despite no change in ACLY mRNA levels (Figure 1C).

To further investigate these findings, we performed immunofluorescence (IF) staining for ACLY and NeuN or β‐III‐tubulin. ACLY was found to be highly colocalized in both the soma and neurites of neurons. In 5×FAD mice, ACLY fluorescence intensity began to decline at 5 months of age and β‐III‐tubulin intensity decreased by 8 months, while NeuN levels remained unchanged (Figure 1D, E). Similar reductions in ac‐tubulin and ACLY levels were observed in the hippocampus of APP/PS1 mice, another widely used AD mouse model (Figure S1B–D). Additionally, fluorescence staining of *post mortem* human brain samples revealed that ACLY was almost entirely colocalized with neurons. However, neuronal ACLY levels were significantly lower in AD brains compared to non‐AD brains (Figure 1F). These findings suggest that neuronal ACLY protein levels are closely associated with the degree of tubulin acetylation. Nevertheless, the physiological and pathological roles of ACLY and ACLY‐mediated tubulin acetylation in the brain remain to be fully elucidated.

### 3.2 Inhibition of ACLY enzyme activity impairs learning and memory in WT mice

**FIGURE 2 alz70919-fig-0002:**
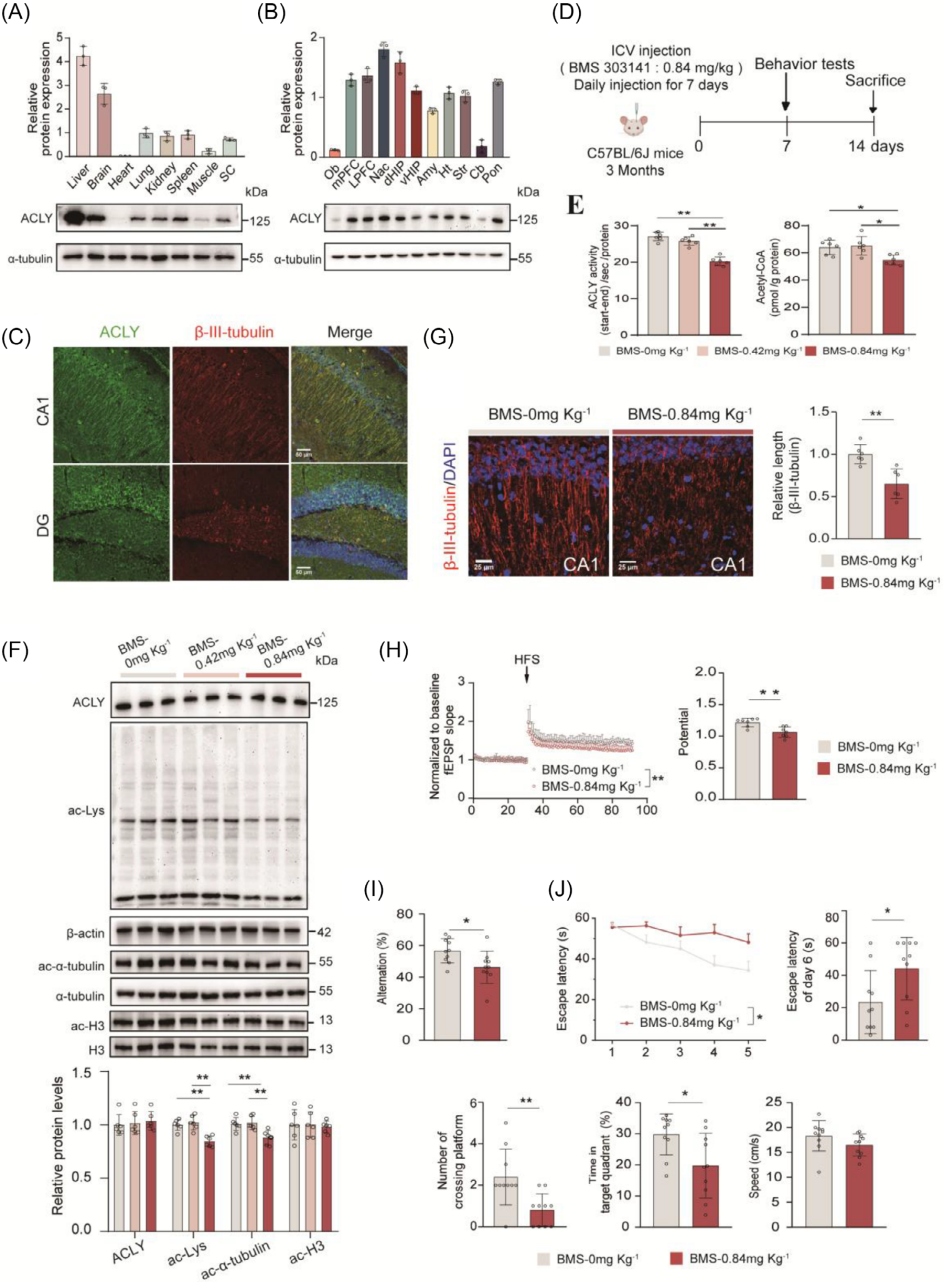
Inhibition of ACLY enzyme activity impaired the learning and memory in WT mice. A, B, Western blotting analysis of ACLY expression across different organs (A) and in different regions of the brain. (B) *n *= 3 mice. (C) IF staining showed the distribution of ACLY (green), β‐III‐Tubulin (red) and DAPI (blue) in the dHip; scale bar = 50 µm; *n *= 3 mice. D, A schematic diagram illustrates the experimental procedure for injecting the ACLY inhibitor into the lateral ventricle. Three‐month‐old C57BL/6J mice were cannulated in the lateral ventricle and allowed to recover for 1 week. After recovery, the ACLY inhibitor BMS‐303141 (0.42 mg kg^−^, 0.84 mg kg^1^) or vehicle was administered daily for 7 days. After the treatment, behavioral tests, electrophysiological recordings, and biochemical analyses were conducted. E, Detection of ACLY enzymatic activity (left) and quantification of acetyl‐CoA level (right) in the dHip of C57BL/6J mice, *n* = 6 mice per group. F, Western blot analysis of ACLY, ac‐Lys, ac‐α‐tubulin/α‐tubulin, ac‐H3/H3 protein levels in the dHip; *n *= 6 mice per group. G, IF staining of β‐III‐Tubulin (red) and DAPI (blue) in hippocampal CA1 region of the mice (left), quantifications of β‐III‐Tubulin positive length (right); scale bar = 25 µm; *n *= 6 slices from three mice per group. H, Normalized EPSC slope in LTP recordings from the CA1 recording electrode. The baseline was stabilized for 30 minutes before HFS induction and the next 60 minutes of recording (left). fEPSPs amplitude quantification during the last 10 minutes of LTP recording (right); *n *= 7 slices from three mice in BMS 0 mg/kg (gray), *n *= 10 slices from three mice in BMS 0.84 mg/kg (red). I, J, Behavioral tests for Y‐maze and MWM. Correct spontaneous alternation rate in a Y‐maze (I). The MWM test was used to assess spatial memory. Escape latency to the platform during the training trials. Latency of first time to enter the target, number of crossings of target, time spent in target quadrant, and the mean swimming speed of mice in the probe trial of MWM (J); *n *= 10 to 11 mice per group. Data are expressed as mean ± standard error of the mean. ^*^ *P *< 0.05, ^**^ *P *< 0.01; One‐way ANOVA with a Tukey multiple comparisons test was used in (E) and (F). One‐way repeated measure ANOVA was used in (J). Two‐tailed unpaired Student *t* test was used in (G), (H), (I), and (J). ACLY, ATP‐citrate lyase; Amy, amygdala; ANOVA, analysis of variance; Cb, the cerebellum; DAPI, 4′,6‐diamidino‐2‐phenylindole; dHIP, dorsal hippocampus; EPSC, excitatory postsynaptic potential; fEPSP, field excitatory postsynaptic potential; HFS, high‐frequency stimulation; Ht, hypothalamus; ICV, intracerebroventricular; IF, immunofluorescence; LPFC, ventrolateral prefrontal cortex; LTP, long‐term potentiation; mPFC, the medial prefrontal cortex; MWM, Morris water maze; Nac, nucleus accumbent; Ob, olfactory bul; Pon, pons; Str, striatum; vHIP, ventral hippocampus; WT, wild type

Acetyl‐CoA, a central metabolite involved in cellular energy production, lipid synthesis, and epigenetic modification, plays a crucial role in cognition and brain function.^44^, ^45^ Our research,^25^ along with studies by Shelley et al. and Eytan Zlotorynski,^24^, ^46^ has demonstrated that ACSS2, which converts acetate into acetyl‐CoA, is vital for cognitive function in both normal and AD conditions. However, the role of ACLY in the brain remains unclear. In this study, we first examined ACLY protein expression across various organs and found it to be most abundant in the liver and brain (Figure 2A). Further analysis revealed that ACLY is widely expressed in brain regions such as the hippocampus and cortex, except the OB and Cb (Figure 2B). IF co‐staining of ACLY with neural cell markers (β‐III‐tubulin, glial fibrillary acidic protein, and ionized calcium‐binding adaptor molecule 1) indicated that ACLY is predominantly enriched in hippocampal neurons, including the cell bodies and processes of neurons (Figure 2C, Figure S2A in supporting information). These findings are consistent with results obtained from primary cultures of different neural cells (Figure S2B).

To investigate the physiological role of neuronal ACLY expression, we first used the small molecule inhibitor BMS‐303141, which targets ACLY.^47^ Treatment with BMS‐303141 at concentrations of 1 and 5 µM significantly reduced ACLY enzyme activity and acetyl‐CoA levels in primary cultured neurons (Figure S2D), without affecting the levels of ACLY and ACSS2 proteins (Figure S2C). We then administered BMS‐303141 into the lateral ventricle of WT C57BL/6J mice (Figure 2D). Continuous administration of BMS‐303141 (0.84 mg/kg daily for 7 days) significantly decreased ACLY enzyme activity and acetyl‐CoA levels in hippocampal tissue (Figure 2E), reduced pan‐acetylation and acetylated tubulin levels, and had no effect on H3 acetylation levels (Figure 2F). Because tubulin acetylation is essential for maintaining microtubule stability,^48^, ^49^ we performed IF staining for β‐III‐tubulin, revealing that BMS‐303141 significantly reduced the length of β‐III‐tubulin in the hippocampal CA1 region (Figure 2G). These findings indicate that inhibition of ACLY enzyme activity downregulates tubulin acetylation and disrupts microtubule stability.[Fig alz70919-fig-0003]

Alterations in memory‐related behavioral functions are closely associated with dysregulated neuronal activity and synaptic plasticity, ultimately leading to the impairment of LTP. To further explore this, we recorded LTP from the stratum radiatum in the CA1 region by stimulating hippocampal Schaffer collaterals. Our findings revealed a significant reduction in field excitatory postsynaptic potentials (fEPSPs) in mice treated with BMS‐303141 (Figure 2H). We then assessed the effects of BMS‐303141 on working memory and spatial learning memory using the Y‐maze and MWM tests. Compared to vehicle‐treated mice, BMS‐303141‐treated mice demonstrated fewer correct rotations in the Y‐maze test (Figure 2I) and increased escape latency to the platform position during both the training (days 1–5) and the probe (day 6) trials. During the testing period, BMS‐303141 treatment significantly reduced the number of platform crossings and the time spent in the target quadrant, without affecting swimming speed (Figure 2J). These results indicate that inhibiting ACLY enzyme activity impairs cognitive function in WT mice. Overall, these data underscore the critical role of brain ACLY activity in maintaining normal cognitive function.

### 3.3 Knockdown of neuronal ACLY in the dorsal hippocampus led to impaired learning and memory in WT mice

**FIGURE 3 alz70919-fig-0003:**
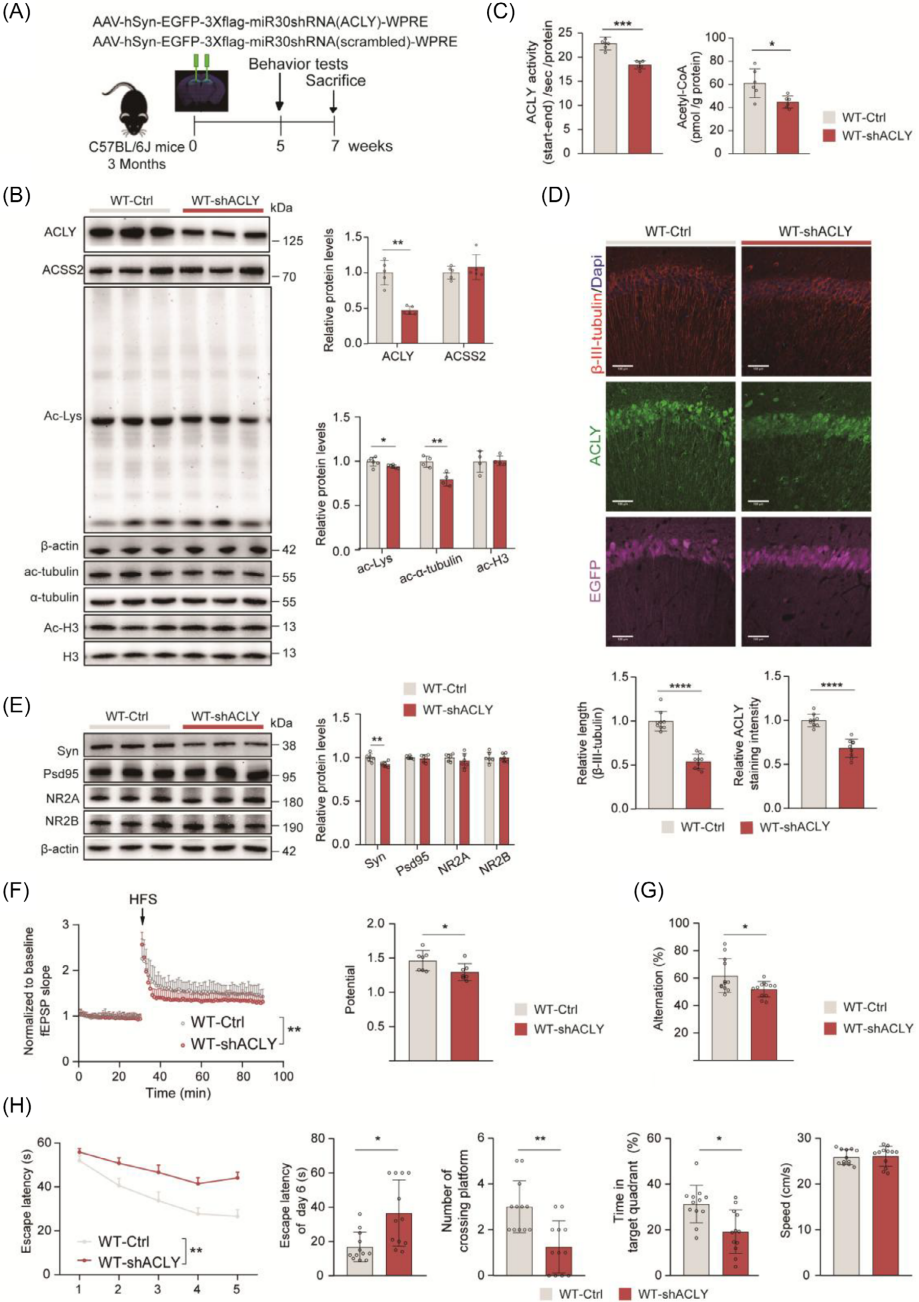
Knockdown of neuronal ACLY in the dHip impaired learning and memory in WT mice. The time schedule for adeno‐associated virus (AAV)‐mediated ACLY knockdown in dorsal hippocampal neurons. B, E, Representative immunoblots and quantitative analyses of dHip lysates for indicated proteins. β‐Actin was used as loading control; *n *= 4 to 6 mice per group in (B); *n *= 6 mice per group in (E). C, Detection of ACLY enzymatic activity (left) and quantification of acetyl‐CoA level (right) in the dHip; *n *= 6 mice per group. D, IF staining of β‐III‐Tubulin (red), ACLY (green), EGFP (purple) and DAPI (blue) in the hippocampal CA1 region (up), quantifications of β‐III‐Tubulin positive length (down, left) and ACLY staining intensity (down, right). Scale bar = 100 µm; *n *= 9 slices from three mice per group. F, Normalized EPSC slope in LTP recordings from the CA1 recording electrode. The baseline was stabilized for 30 minutes before HFS induction and the next 60 minutes of recording (left). fEPSP amplitude quantification during the last 10 minutes of LTP recording (right); *n *= 7 slices from three mice in WT‐Ctrl (gray), *n *= 9 slices from three mice in WT‐sh*ACLY* (red). G−H Behavioral tests for Y‐maze and MWM. Correct spontaneous alternation rate in a Y‐maze (G). Escape latency to the platform during the training trials. Latency of first time to enter the target, number of times crossing target, time spent in target quadrant, and the mean swimming speed of mice in the probe trial of MWM (H); *n *= 10 to 12 mice per group. Data were expressed as the mean ± standard error of the mean. ^*^ *P *< 0.05, ^**^ *P *< 0.01, ^***^ *P *< 0.001, ^****^ *P *< 0.0001, Two‐tailed unpaired Student *t* test was used except for one‐way repeated measure analysis of variance was used in (H). ACLY, ATP‐citrate lyase; DAPI, 4′,6‐diamidino‐2‐phenylindole; dHip, dorsal hippocampus; EGFP, enhanced green fluorescent protein; EPSC, excitatory postsynaptic potential; fEPSP, field excitatory postsynaptic potential; HFS, high‐frequency stimulation; IF, immunofluorescence; LTP, long‐term potentiation; MWM, Morris water maze; WT, wild type

To further clarify the importance of neuronal ACLY expression in hippocampal‐mediated spatial learning and memory, we downregulated ACLY levels in the dHip of 3‐month‐old C57BL/6J mice by injecting an adeno‐associated virus (AAV) carrying sh*ACLY* (Figure 3A). Compared to control mice, sh*ACLY*‐treated mice exhibited significantly reduced levels of ACLY protein, enzyme activity, and acetyl‐CoA, while the levels of ACSS2 protein remained unaffected (Figure 3B–C). Consistent with inhibition of ACLY enzyme activity, knockdown of neuronal ACLY decreased pan‐acetylation and acetylated α‐tubulin levels (Figure 3B) and shortened β‐III‐tubulin (Figure 3D), with no effect on H3 acetylation levels in the dorsal hippocampus. Additionally, analysis of synaptic‐associated protein expression revealed that ACLY knockdown significantly reduced the levels of synaptophysin, a presynaptic marker, while the expression of postsynaptic markers such as PSD95, NMDAR2A, and NMDAR2B remained unchanged (Figure 3E). Electrophysiological recordings further indicated that ACLY knockdown significantly compromised LTP in WT mice (Figure 3F). Consistently, ACLY knockdown in the dHip reduced the performance of WT mice in both Y‐maze (Figure 3G) and MWM maze behavioral tests (Figure 3H). These results suggest that neuronal ACLY expression is essential for learning and memory in mice.

### 3.4 Knockdown of neuronal ACLY exacerbates dystrophic neurites, impairs autophagic–lysosomal flux, and accelerates Aβ deposition in the early stage of 5×FAD mice

**FIGURE 4:**
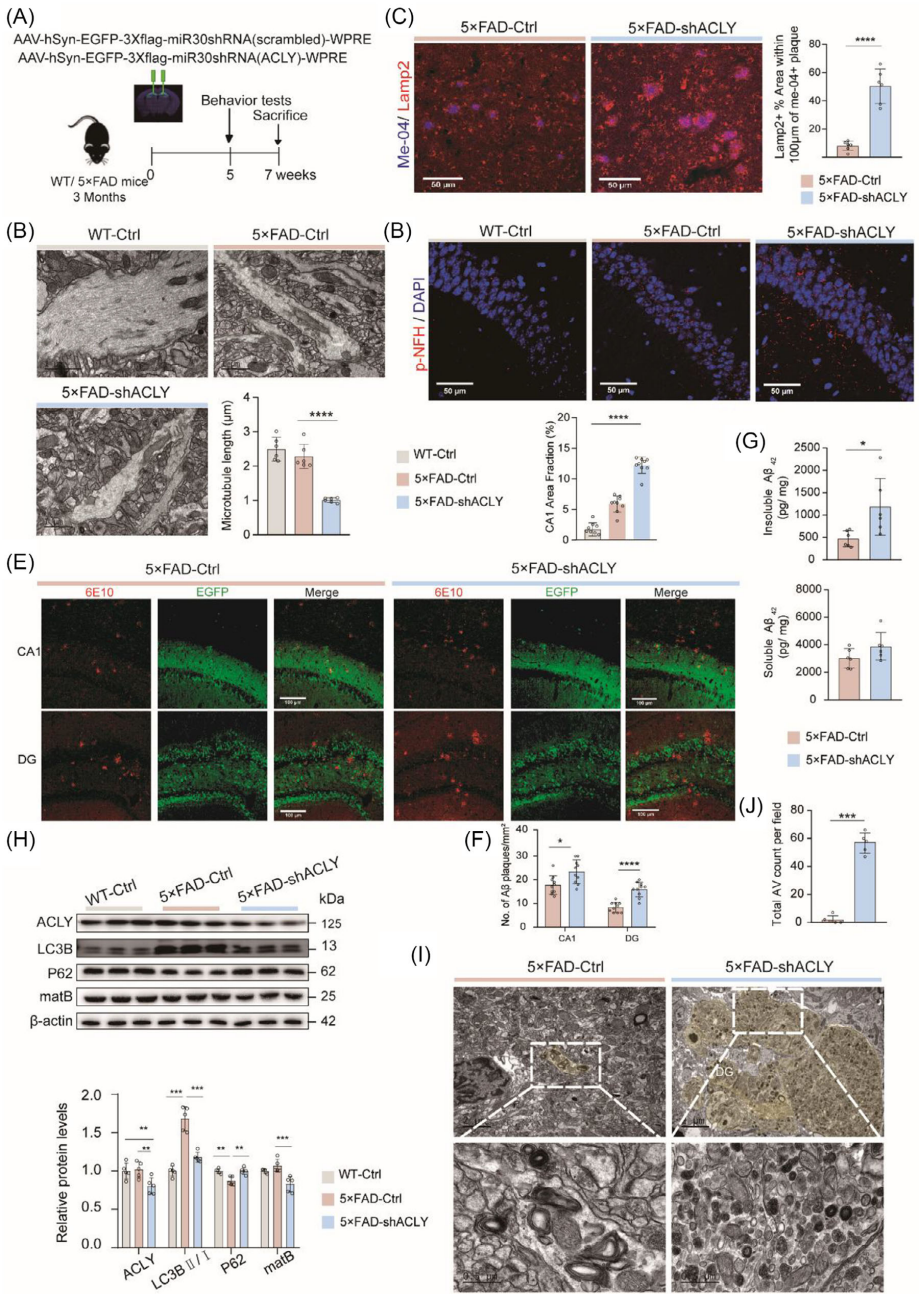
Knockdown of neuronal ACLY exacerbated amyloid pathology and DNs in the early AD stage. A, Schematic of the experimental work flow. B, Representative TEM images and quantification of microtubule in CA1. Gray blue areas indicate microfilament; scale bar = 1 µm; *n *= 3 mice pre group. C−F, Representative images and quantification of the dorsal hippocampus immunostained with indicated antibody; scale bars = 50 µm in (C) and (D); scale bars = 100 µm in E; *n *= 6 to 10 slices from three mice pre group. G, ELISA analysis of the level of insoluble Aβ_42_ and soluble Aβ_42_ in hippocampal lysates; *n *= 6 mice pre group. H, Representative immunoblots and quantitative analyses of hippocampal lysates for indicated proteins. β‐actin was used as loading control; *n *= 5 mice per group. I−J, Representative TEM images and quantitative analyses of autophagic lysosomes in CA1. Yellow areas indicate for autophagic lysosomes; scale bar = 2 µm, 0.5 µm; *n *= 3 mice pre group. Data are expressed as mean ± standard error of the mean. ^*^ *P *< 0.05, ^**^ *P *< 0.01, ^***^ *P *< 0.001, ^****^ *P *< 0.0001. One‐way analysis of variance with a Tukey multiple comparisons test was used in (B), (D), and (H). The unpaired two‐tailed *t* test was used in (C), (F), (G), and (J). Aβ, amyloid beta; ACLY, ATP‐citrate lyase; AD, Alzheimer's disease; DN, dystrophic neurite; ELISA, enzyme‐linked immunosorbent assay; TEM, transmission electron microscope

To investigate the role of neuronal ACLY in the early pathological progression of AD, we injected AAV carrying sh*ACLY* into the dHip of 3‐month‐old 5×FAD mice (Figure 4A). Compared to 5×FAD‐Ctrl mice, the 5×FAD‐sh*ACLY* mice exhibited significantly reduced ACLY expression and enzyme activity, as well as lower acetyl‐CoA levels in the dHip (Figure S3A, B in supporting information). Consistent with the effects of ACLY knockdown in WT mice (Figure 3), we also observed impaired tubulin acetylation (Figure S3C), shortened β‐III‐tubulin length (Figure S3D), reduced levels of presynaptic protein synaptophysin (Figure S3E), and fewer synapses per unit area (Figure S3F) in 5×FAD‐sh*ACLY* mice. Furthermore, transmission electron microscopy (TEM) revealed that microtubules in 5×FAD‐sh*ACLY* group were irregular in shape and short in length (Figure 4B). Lamp2‐positive DNs were more pronounced in 5×FAD‐sh*ACLY* mice (Figure 4C). In addition, an increase in pNF‐H‐positive bulb‐like swollen axons—an indicator of axonal deterioration due to Aβ plaque accumulation in the brains of AD patients,^50^, ^51^ was observed in 5×FAD‐Ctrl mice compared to WT‐Ctrl mice, and this increase was further exacerbated in 5×FAD‐sh*ACLY* mice (Figure 4D). These findings suggest that knockdown of neuronal ACLY not only disrupts microtubule stability but also aggravates neurite damage in response to amyloid plaque toxicity.

Comparing Aβ deposition in the dHip of 5×FAD mice, we observed that the knockdown of neuronal ACLY significantly increased the area of Aβ plaques (Figure 4E, F). We then analyzed amyloids levels in the dHip of 5×FAD using ELISA. The levels of insoluble Aβ_42_ were significantly elevated in 5×FAD‐sh*ACLY* group, and no significant changes were observed in soluble Aβ_42_ (Figure 4G). Given that the autophagic–lysosomal pathway is a crucial mechanism for amyloid degradation, we next examined the expression of relevant markers through immunoblotting. The level of the autophagic marker LC3‐II, which was elevated in 5×FAD mice compared to WT mice, was decreased in 5×FAD‐sh*ACLY* mice. Conversely, the level of p62, a marker of autophagic flux, decreased in 5×FAD mice compared to WT mice but increased in 5×FAD‐sh*ACLY* mice. Additionally, the level of mature cathepsin B, a lysosomal protease, was reduced in 5×FAD‐sh*ACLY* mice (Figure 4H). Furthermore, TEM revealed numerous AVs in the dorsal CA1 of 5×FAD‐sh*ACLY* mice (Figure 4I, J). These findings indicate that knockdown of neuronal ACLY impairs autophagosome–lysosome flux in 5×FAD mice. Overall, these data suggest that decreased ACLY expression promotes Aβ deposition, likely by impairing intracellular degradation of Aβ.

### 3.5 Upregulation of neuronal ACLY improves microtubule stability and reduces DNs in the late stages of 5×FAD mice

**FIGURE 5:**
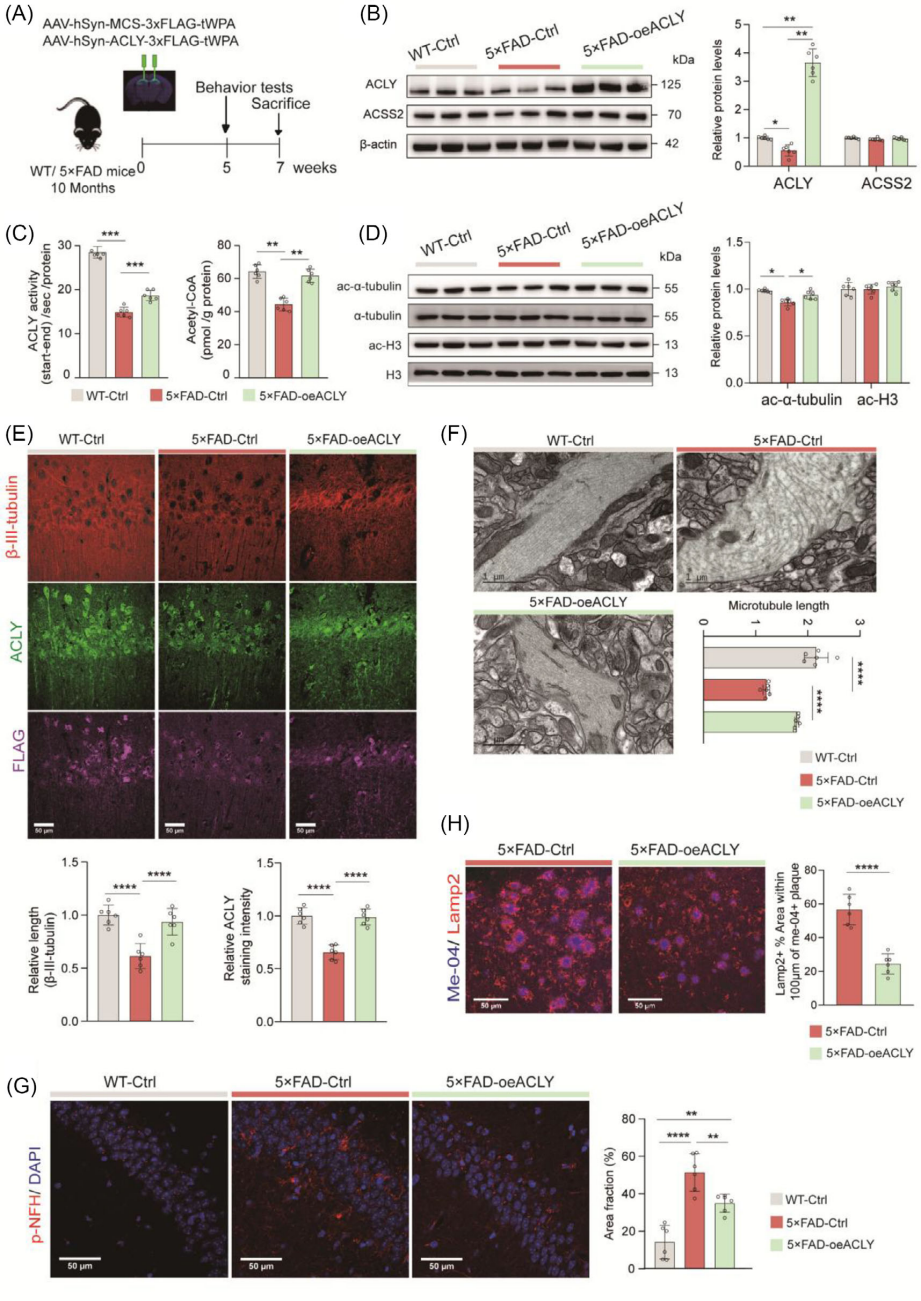
Upregulation of neuronal ACLY improved microtubule stability and decreased dystrophic neurites in the late Alzheimer's disease stage. A, Schematic of the experimental work flow. B, D, Representative immunoblots and quantitative analyses of dorsal hippocampal lysates for indicated antibody; *n *= 6 mice per group. C, Detection of ACLY enzymatic activity (left) and quantification of acetyl‐CoA level (right) in the dHip lysates; *n *= 5−6 mice per group. E, G, H, Representative images and quantification of the dHip immunostained with indicated antibody; scale bar = 50 µm in (E), (G), and (H); *n *= 6 slices from threee mice per group. F, Representative TEM images and quantification of microtubule in CA1. Gray blue indicates microfilaments; scale bar = 1 µm; *n *= 3 mice per group. Data are expressed as mean ± standard error of the mean. ^*^ *P *< 0.05, ^**^ *P *< 0.01, ^****^ *P *< 0.0001. One‐way analysis of variance with a Tukey multiple comparisons test was used, except for two‐tailed unpaired Student *t* test was used in H. ACLY, ATP‐citrate lyase; dHip, dorsal hippocampus; TEM, transmission electron microscope; WT, wild type

Given the critical role of neuronal ACLY expression in maintaining microtubule stability and regulating Aβ deposition, we next examined the impact of elevated ACLY levels on neuropathological progression in 10‐month‐old 5×FAD mice (Figure 5A). As expected, a significant increase in ACLY expression, ACLY enzyme activity, acetyl‐CoA levels, and acetylated α‐tubulin levels was observed in the 5×FAD‐oe*ACLY* group (Figure 5B–D). Subsequent IF staining revealed that upregulation of ACLY markedly improved the length of β‐III‐tubulin in 5×FAD mice (Figure 5E). TEM further demonstrated that the irregular and shortened microtubule structures were ameliorated in the 5×FAD‐oe*ACLY* group (Figure 5F). Additionally, pNF‐H‐positive bulb‐like swollen axons (Figure 5G) and lamp2‐positive DNs (Figure 5H) were reduced in 5×FAD‐oe*ACLY* mice compared to 5×FAD‐Ctrl mice. These findings suggest that upregulation of neuronal ACLY not only restores microtubule stability but also mitigates neurite damage in response to amyloid plaque toxicity in late‐stage 5×FAD mice.

### 3.6 Upregulation of neuronal ACLY enhances autophagic–lysosomal flux and alleviates Aβ deposition in the late stage of 5×FAD mice

**FIGURE 6:**
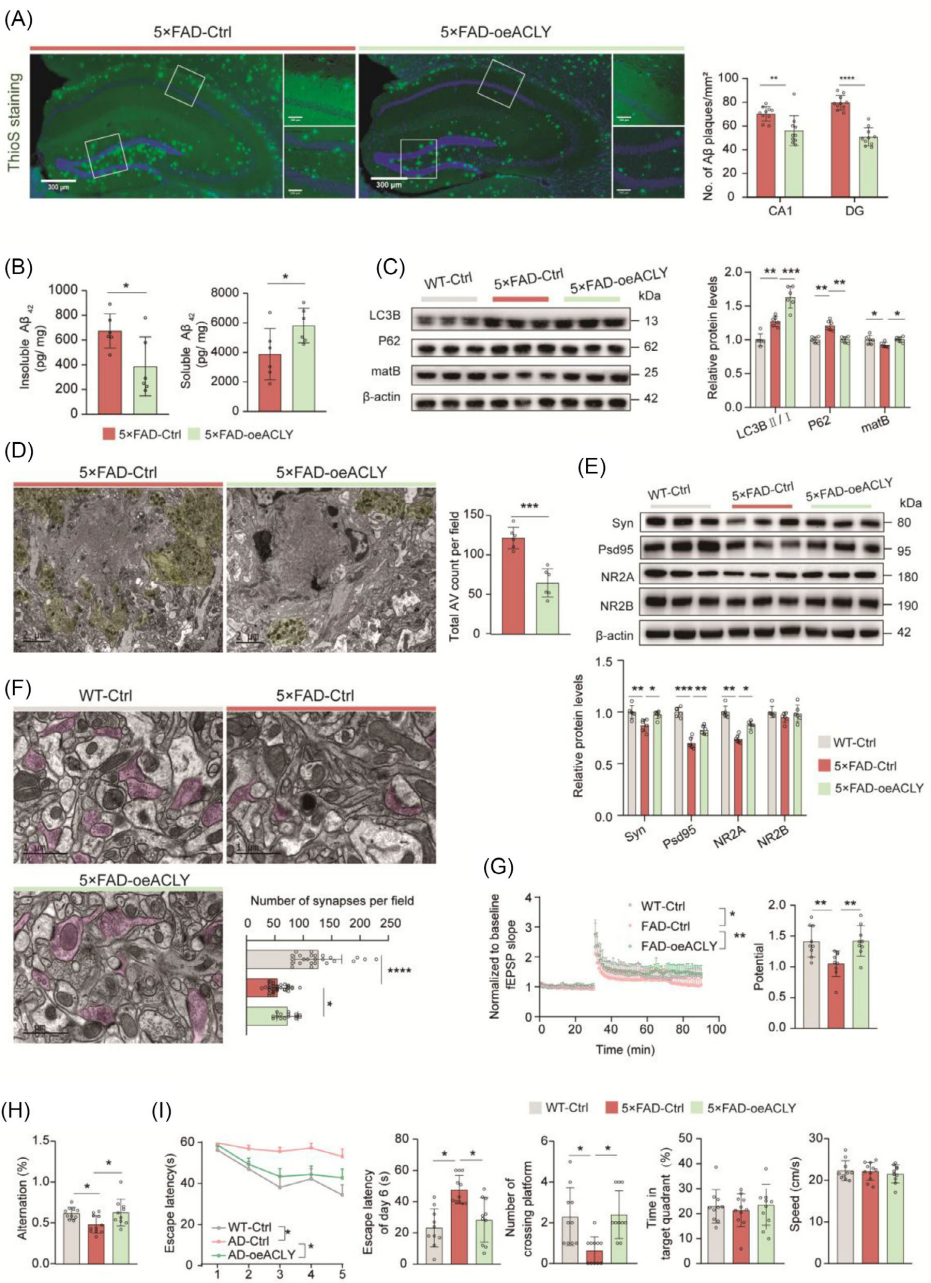
Upregulation of neuronal ACLY enhanced autophagic–lysosomal flux, alleviates Aβ deposition and improved cognition in the late AD stage. A, Representative images and quantification of Aβ deposition in the dHip with Thioflavin S (ThioS) staining; scale bar = 300 µm, 100 µm; *n *= 10 slices from three mice per group. B, ELISA analysis of the level of insoluble Aβ_42_ and soluble Aβ_42_ in hippocampal lysates; *n *= 6 mice pre group. C, E, Representative immunoblots and quantitative analyses of dorsal hippocampal lysates for indicated proteins. β‐Actin was used as loading control; *n *= 6 mice pre group, normalized by WT‐Ctrl. D, F, Representative TEM images and quantitative analyses. D, Autophagic lysosomes in CA1. Yellow areas indicate autophagic lysosomes; scale bar = 2 µm; *n *= 3 mice. F, Typical synaptic structure of hippocampus CA1. Pink areas indicate presynaptic regions; scale bar = 1 µm, *n *= 3 mice. G, Normalized EPSC slope in LTP recordings from the CA1 recording electrode. The baseline was stabilized for 30 minutes before HFS induction and the next 60 minutes of recording (left). fEPSP amplitude quantification during the last 10 minutes of LTP recording (right); *n *= 9 slices from three mice per group. H, I, Behavioral tests for Y‐maze and MWM. Correct spontaneous alternation rate in a Y‐maze (H), *n *= 10 mice per group. Escape latency to the platform during the training trials. Latency of first time to enter the target, number of times crossing target, time spent in target quadrant, and the mean swimming speed of mice in the probe trial of MWM (I); *n *= 10 to 11 mice per group. Data were expressed as the mean ± standard error of the mean. ^*^ *P *< 0.05, ^**^ *P *< 0.01, ^****^ *P *< 0.0001. The unpaired two‐tailed *t* test were used in (A), (B), and (D). One‐way analysis of variance with a Tukey multiple comparisons test were used in (C) and (E–I). Aβ, amyloid beta; ACLY, ATP‐citrate lyase; AD, Alzheimer's disease; DN, dystrophic neurite; ELISA, enzyme‐linked immunosorbent assay; EPSC, excitatory postsynaptic potential; fEPSP, field excitatory postsynaptic potential; HFS, high‐frequency stimulation; LTP, long‐term potentiation; MWM, Morris water maze; TEM, transmission electron microscope; WT, wild type

We then assessed Aβ deposition using Thioflavin S (ThioS) staining and found that Aβ deposition was significantly reduced in the 5×FAD‐oe*ACLY* group compared to the 5×FAD‐Ctrl mice (Figure 6A). Furthermore, ELISA analysis revealed a decrease in insoluble Aβ_42_ and an increase in soluble Aβ_42_ after *ACLY* overexpression in the dHip of 5×FAD mice (Figure 6B). In the late stages of AD, Aβ deposition is primarily influenced by an impaired autophagy pathway.^52^ Upregulated LC3‐II, downregulated mature lysosomal cathepsin B expression, and increased p62 levels in the dHip of 5×FAD‐Ctrl mice were restored in 5×FAD‐oe*ACLY* mice (Figure 6C). Additionally, the unique pattern termed “Panthos” characterized by profuse AVs forming large membrane blebs that create flower‐like perikaryal rosettes in more compromised but still intact neurons,^53^, ^54^ frequently observed in 5×FAD‐Ctrl mice, was significantly decreased in the 5×FAD‐oe*ACLY* mice (Figure 6D). These results suggest that upregulation of neuronal ACLY not only reduces Aβ deposition but also alleviates severe impairment of autophagic–lysosomal function in late‐stage 5×FAD mice.

### 3.7 Upregulation of neuronal ACLY enhances synaptic plasticity and improves cognitive function in the late stage of 5×FAD mice

To further investigate the role of ACLY in cognitive protection in 10‐month‐old 5×FAD mice, we performed Western blot analysis of synaptic‐associated proteins. The results showed that the levels of Syn, PSD95, and NR2A were decreased in 5×FAD‐Ctrl mice compared to WT‐Ctrl mice, while these levels were increased in 5×FAD‐oe*ACLY* mice (Figure 6E). TEM further revealed that restoring neuronal ACLY levels significantly increased the number of synapses in the dorsal hippocampal CA1 region (Figure 6F). We next measured LTP by recording fEPSPs from the stratum radiatum in CA1 after stimulation of hippocampal Schaffer collaterals. A significant increase in fEPSPs was observed in 5×FAD‐oe*ACLY* mice compared to 5×FAD‐Ctrl mice (Figure 6G). Consistent with these findings, both Y‐maze (Figure 6H) and MWM maze behavioral tests (Figure 6I) showed that the reduced performance observed in 5×FAD‐Ctrl mice was rescued in 5×FAD‐oe*ACLY* mice. These results suggest that upregulation of neuronal ACLY enhances synaptic plasticity and alleviates cognitive impairment in late‐stage 5×FAD mice.

### 3.8 ACLY regulates autophagic–lysosomal flux and lysosomal acidification in N2a‐APP695 cells

**FIGURE 7:**
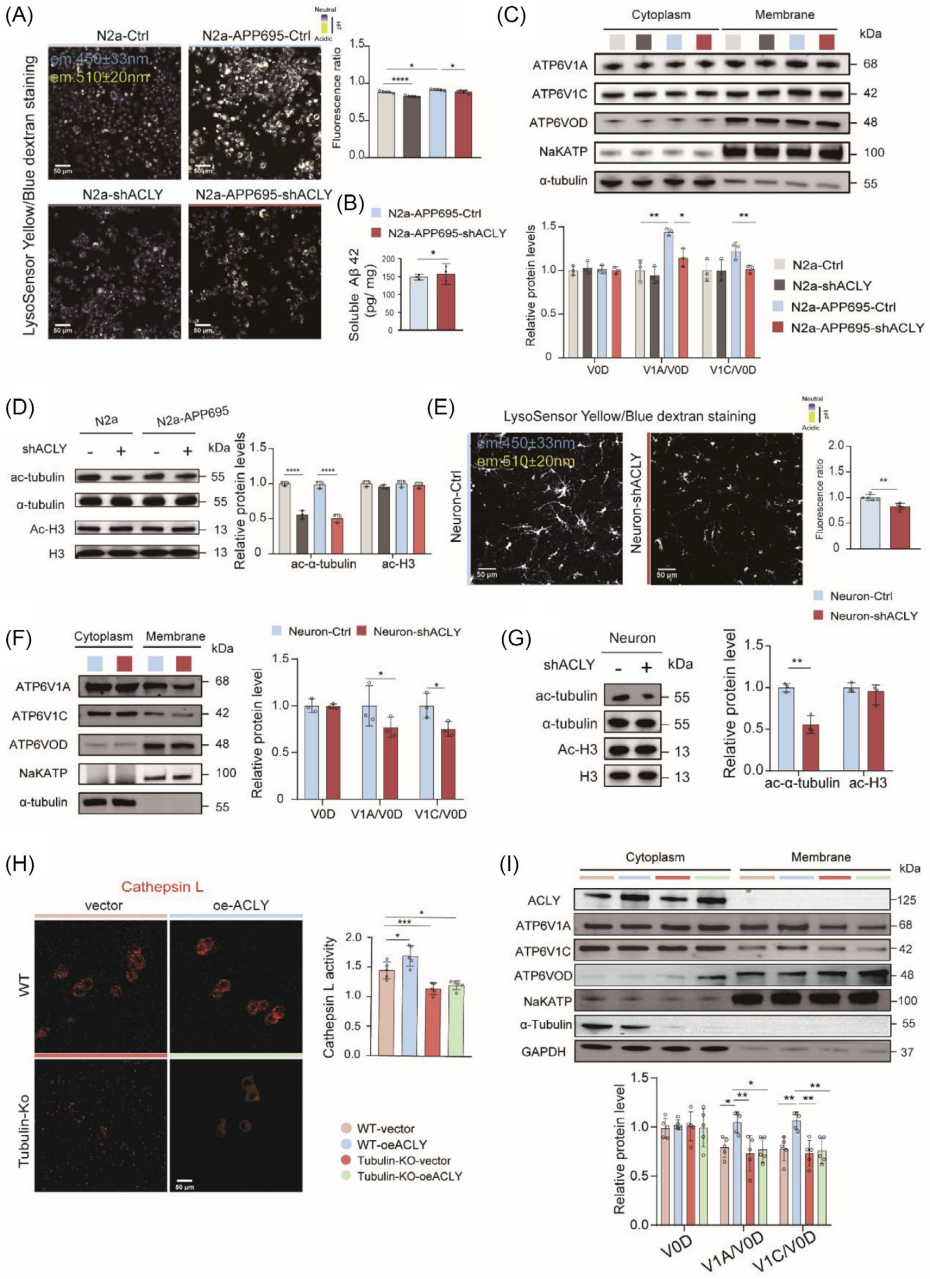
ACLY regulates assembly of the lysosomal V‐ATPase holoenzyme via α‐tubulin acetylation in N2a cells. A, E, Determination of lysosomal pH with the ratio metric probe LysoSensor Yellow/Blue dextran. Scale bar = 50 µm. *n* = 5 per group. B, ELISA analysis the level of soluble Aβ_42_ in cell lysates; *n *= 3 per group. C, F, I, Representative immunoblots and quantitative analyses of the V‐ATPase V1‐V0 holoenzyme in cells. Na‐K‐ATPase and α‐tubulin are used as markers for membrane proteins and cytoplasm protein, respectively; *n *= 3 to 5 per group. D, G, Representative immunoblots and quantitative analysis of cells lysates for indicated proteins. β‐actin was used as loading control. Normalized by N2a‐Ctrl group or neuron‐Ctrl; *n *= 3 per group. H, Representative images and quantification of Cathepsin L activity with MagicRed in wild‐type (WT) N2a cells and tubulin‐KO N2a cells after fasting for 1 hour; scale bar = 50 µm; *n* = 5 per group. Data are expressed as mean ± standard error of the mean. ^*^ *P *< 0.05, ^**^ *P *< 0.01, ^***^ *P *< 0.001, ^****^ *P *< 0.0001. Two‐way analysis of variance with a Tukey multiple comparisons was used in (A), (C), (D), (H), and (I). The unpaired two‐tailed *t* test were used in (B), (E), (F), and (G). Data are representative of three independent experiments. Aβ, amyloid beta; ACLY, ATP‐citrate lyase; ELISA, enzyme‐linked immunosorbent assay; V‐ATPase, vacuolar‐adenosine triphosphatase; WT, wild type

To further elucidate the mechanism by which ACLY influences autophagic–lysosomal flux in AD mice, we used the N2a‐sw‐APP695 cell line, a classical model in which neurons produce Aβ. Western blot analysis revealed that N2a‐sw‐APP695 cells exhibited enhanced autophagic–lysosomal flux compared to N2a cells, characterized by upregulation of LC3B and mature cathepsin B (maCatB), and downregulation of p62, while ACLY levels remained unchanged (Figure S4A in supporting information). Consistent with in vivo observations, *ACLY* knockdown led to a decline in autophagic–lysosomal flux in N2a‐sw‐APP695 cells, evidenced by downregulation of LC3B and maCatB and an increase in p62 (Figure S5A in supporting information), with the opposite effect observed upon *ACLY* overexpression (Figure S5B). Maintaining a mildly acidic pH is crucial for lysosomal enzyme activity.^11^, ^55^ Lysosomal acidification was assessed using LysoSensor Yellow/Blue probes, which offer dual excitation and emission spectra dependent on pH. This probe has a pKa of ≈ 4.2, producing predominantly yellow fluorescence in acidic organelles and blue fluorescence in less acidic organelles, thereby providing a reliable ratiometric measurement of lysosomal pH within a range of 3.5 to 6.0.^56^ Compared to N2a cells, N2a‐sw‐APP695 cells displayed stronger yellow fluorescence and weaker blue fluorescence (Figure S4B). Importantly, *ACLY* knockdown resulted in stronger blue fluorescence and weaker yellow fluorescence in N2a‐sw‐APP695 cells (Figure 7A), while *ACLY* overexpression had the opposite effect (Figure S5C). Not surprisingly, Aβ_42_ levels in the lysates showed a consistent change with ACLY expression (Figure 7B, Figure S5D). These findings indicate that ACLY levels influence lysosomal acidification and autophagic–lysosomal flux in N2a‐sw‐APP695 cells.

### 3.9 ACLY regulates the assembly of the lysosomal V‐ATPase holoenzyme through α‐tubulin acetylation

Lysosomal acidification is primarily regulated by the V‐ATPase complex, which facilitates proton translocation between the cytosol and the lysosomal lumen.^57^, ^58^ The V‐ATPase complex consists of the V1 peripheral domain and the V0 integral membrane domain, each composed of multiple subunits. The cytosolic V1 subunits must associate with the membrane‐embedded V0 subunits to form the V1‐V0 holoenzyme, which constitutes a functional proton pump.^12^, ^59^ To investigate the mechanism by which ACLY regulates lysosomal acidification, we first isolated the membrane fraction of cells and quantified the abundance of membrane‐associated V1 subunits V1A and V1C relative to the V0 subunit V0D, as an indicator of assembled V1‐V0 holoenzymes. The levels of V0D in the membrane fractions were comparable after normalization against the membrane marker Na‐K‐ATPase (Figure 7C, Figure S5E), suggesting that the density of the V‐ATPase V0 domain on lysosomes was unaffected by APP695 expression and ACLY levels. Importantly, *ACLY* knockdown led to a decrease in V‐ATPase V1‐V0 holoenzymes (Figure 7C), while ACLY overexpression had the opposite effect (Figure S5E). These findings indicate that ACLY levels influence the assembly of the V‐ATPase holoenzyme, which in turn regulates lysosomal acidification.

Microtubule stability is essential for the assembly and dissociation of lysosomal V‐ATPase subunits,^60^ as well as for lysosomal retrograde transport in neuronal axons.^61^ α‐Tubulin, a crucial component of microtubules, plays a significant role in regulating V‐ATPase holoenzyme assembly through its acetylation at Lys40.^62^, ^63^ Notably, we observed that *ACLY* knockdown led to a decrease in α‐tubulin acetylation (Figure 7D), while *ACLY* overexpression had the opposite effect (Figure S5F), consistent with in vivo results (Figures 3B, 5D). Furthermore, in primary cultured neurons with lentivirus‐mediated *ACLY* knockdown, ACLY depletion significantly impaired lysosomal acidification, decreased the level of V‐ATPase V1‐V0 holoenzyme and α‐tubulin acetylation (Figure 7E–G).

Next, to investigate the essential role of tubulin in the assembly of lysosomal V‐ATPase subunits and its enzyme function mediated by ACLY, we constructed a tubulin knockout (KO) N2a cell line. After fasting for 1 hour, the activity of Cathepsin L in tubulin‐KO cells was significantly lower than that in WT cells. Notably, the phenomenon observed in WT cells—where overexpression of *ACLY* markedly enhanced Cathepsin L activity—was completely absent in tubulin‐KO cells (Figure 7H). Furthermore, compared to WT cells, WT‐oeACLY cells showed a significant increase in the levels of V1A and V1C in the membrane fraction. However, this effect was entirely absent in tubulin‐KO cells (Figure 7I).

**FIGURE 8:**
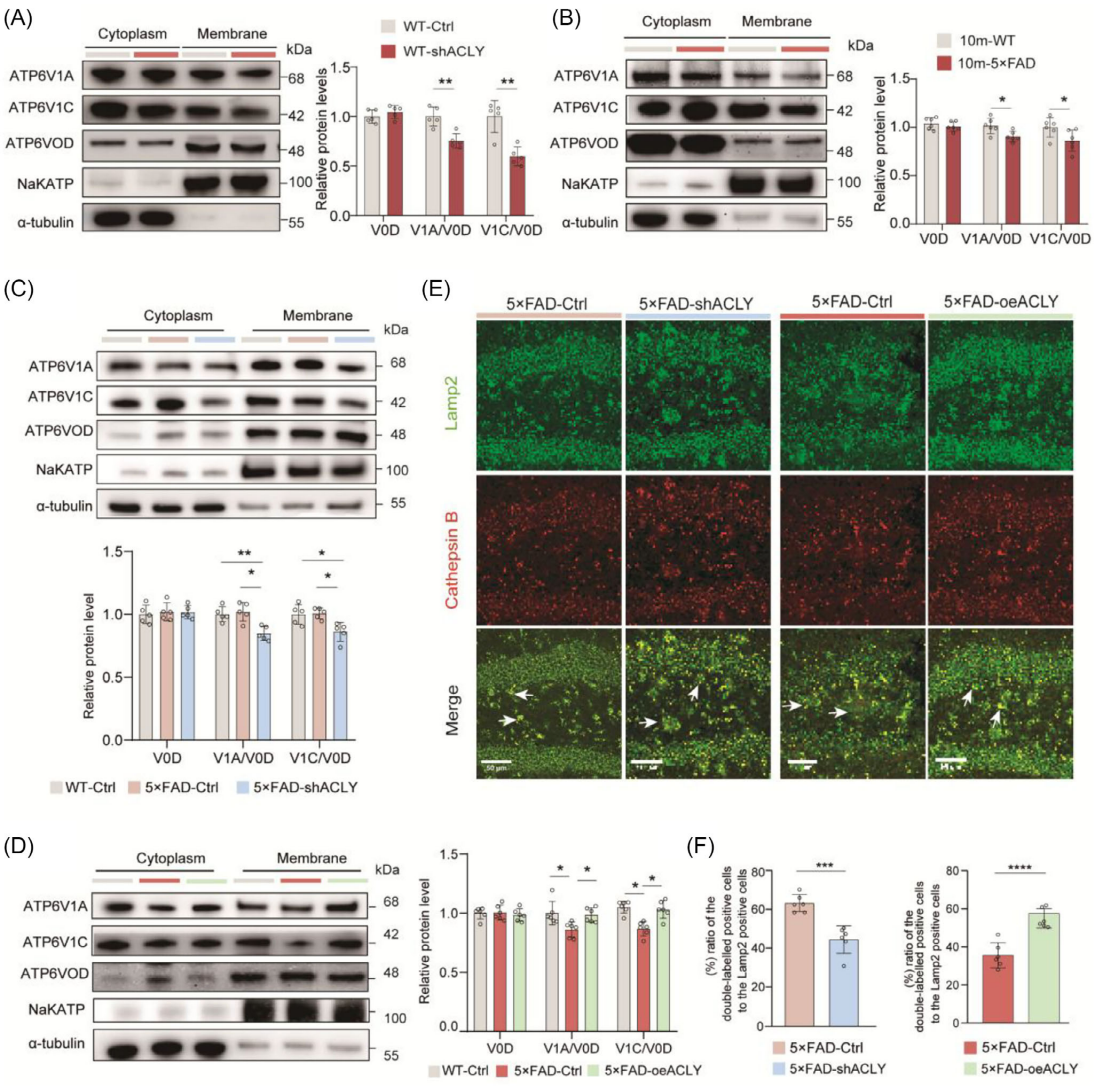
ACLY regulates assembly of the lysosomal V‐ATPase holoenzyme in vivo. A−D, Representative immunoblots and quantitative analyses of dorsal hippocampal lysates for the V‐ATPase V1‐V0 holoenzyme. Na‐K‐ATPase and α‐tubulin are used as markers for membrane proteins and cytoplasm protein, respectively; *n *= 5 to 6 mice per group. E−F, Representative images (E) and quantification (F) of the dorsal hippocampus immunostained with indicated antibody for Cathepsin B enzymatic activity. The white arrows indicate representative staining. Scale bar = 50 µm; *n* = 6 slices from three mice per group. Data are expressed as mean ± standard error of the mean. ^*^ *P *< 0.05, ^**^ *P *< 0.01, ^***^ *P *< 0.01, ^****^ *P *< 0.0001. Unpaired two‐tailed *t* tests were used in (A), (B), and (F). One‐way analysis of variance with a Tukey multiple comparisons test were used in (C) and (D). ACLY, ATP‐citrate lyase; V‐ATPase, vacuolar‐adenosine triphosphatase

Finally, we examined the effect of neuronal ACLY levels on the assembly of the V‐ATPase holoenzyme in the dorsal hippocampus. Compared to WT‐Ctrl mice, WT‐sh*ACLY* mice exhibited a significant decrease in the levels of V1A and V1C in the membrane fraction (Figure 8A). Importantly, in 10‐month‐old 5×FAD mice, the levels of V1A and V1C in the lysosomal membrane were markedly reduced compared to littermate controls (Figure 8B). Knockdown of neuronal *ACLY* in 3‐month‐old 5×FAD mice also led to a noticeable reduction in V1A and V1C levels in the lysosomal membrane (Figure 8C), with the opposite effect observed in 10‐month‐old 5×FAD mice (Figure 8D). Additionally, we found that *ACLY* knockdown in 3‐month‐old 5×FAD mice significantly reduced Cathepsin B levels in the lysosomes, enhancing *ACLY* expression in 10‐month‐old 5×FAD mice obviously increased lysosomal Cathepsin B activity (Figure 8E, F).

Taken together, these findings suggest that ACLY levels influence lysosomal function through α‐tubulin acetylation, which mediates the assembly of the V‐ATPase holoenzyme.

## 4 DISCUSSION

This study assigns ACLY a previously unrecognized role as key metabolic enzyme that not only influences cognition but also regulates Aβ pathology in the pathogenesis of AD. First, neuronal ACLY levels in the hippocampus are essential for maintaining learning and memory through α‐tubulin acetylation‐mediated microtubule stability. Second, we observed a progressive decline in ACLY levels in AD neurons and demonstrated that neuronal ACLY deficiency aggravates dystrophic neurites, exacerbates Aβ deposition, and impairs autophagic–lysosomal flux in the early stages of AD. Importantly, these pathological processes can be significantly alleviated by increasing ACLY levels in the later stages of the disease. Mechanistically, ACLY regulates the assembly of the lysosomal V‐ATPase holoenzyme via α‐tubulin acetylation, which, in turn, affects lysosomal acidification. Overall, this study demonstrates that reduced neuronal ACLY levels in the AD brain leads to a decrease in acetylated α‐tubulin and microtubule stability, thereby impairing the assembly of V‐ATPase on the lysosomal membrane, reducing lysosomal acidification, and exacerbating Aβ pathology. These findings enhance our understanding of AD pathogenesis and suggest new therapeutic approaches targeting neuronal ACLY levels and its enzymatic activity.

In the present study, ACLY is predominantly expressed in neurons, with robust localization to neuronal soma and axons (Figures 1, 2, and Figure S2A, B), consistent with the findings of Even et al., which reported that ACLY is distributed on vesicles in cultured neurons.^43^ Interestingly, altering neuronal ACLY levels or enzyme activity affected only tubulin acetylation, not histone acetylation, suggesting that ACLY is primarily involved in the acetylation of cytoplasmic proteins in neurons. This finding contrasts with previous reports that ACLY‐mediated histone acetylation is predominant in immortalized cell lines,^64^ macrophages,^65^ or astrocytes.^66^ Moreover, it aligns with earlier studies indicating that histone acetylation regulation in neurons primarily relies on ACSS2‐derived nuclear acetyl‐CoA.^24^, ^25^

The functional activity of microtubules is extensively regulated by post‐translational modifications, particularly the acetylation of their α subunits.^67^ Tubulin acetylation modulates axonal transport by facilitating the recruitment of molecular motors to microtubules and the loading of motile vesicles onto these motors.^68^ ^−^ ^71^ In addition to the Elongator complex,^43^, ^69^ the acetylation at lysine 40 (K40) of α‐tubulin is dynamically regulated by acetyltransferases, such as ATAT1^70^ and MEC‐17,^68^ and two histone deacetylase‐related enzymes, HDAC6^41^ and SIRT2.^72^ In the present study, we found that ACLY is enriched in neuronal processes and produces acetyl‐CoA, thereby providing acetyl groups for α‐tubulin. More importantly, this is essential for maintaining synaptic plasticity and cognition. Previous studies have reported that targeting HDAC6 alleviates the phenotypes of multiple neurodegenerative mouse models,^73^ including AD,^35^, ^40^, ^74^ amyotrophic lateral sclerosis (ALS),^75^ and Charcot‐Marie‐Tooth disease (CMT2D).^76^, ^77^ In our study, ACLY—which supplies the direct substrate for tubulin acetylation—exhibited an early age‐dependent decrease, unlike regulatory enzymes such as ATAT1 and HDAC6 (Figure 1C−E and Figure S1). Although the decline in ACLY levels occurs later than cerebral Aβ deposition, our findings demonstrate that ACLY knockdown in 3‐month‐old 5×FAD mice exacerbates key pathological features, whereas its overexpression at a later disease stage confers significant rescue. These results strongly support the role of ACLY as an important modulator of disease progression. We propose a feed‐forward cycle in which Aβ accumulation leads to ACLY downregulation, which in turn accelerates synaptic dysfunction and Aβ deposition. Whether ACLY plays an initiating role in sporadic AD remains an open question, underscoring the need for future studies using models that represent the more common form of this disease.

The autophagic–lysosomal pathway is crucial for maintaining cellular homeostasis by removing cytotoxic protein aggregates, long‐lived proteins, dysfunctional organelles, and pathogens.^20^, ^78^, ^79^ Beyond the importance of endolysosomal biogenesis,^5^, ^80^, ^81^ dysfunction and mistrafficking of organelles within the autophagy and endosomal–lysosomal pathways are implicated in neurodegenerative diseases.^10^, ^17^, ^82^, ^83^ Notably, targeting faulty lysosomal acidification dependent on V‐ATPase, offers a novel approach for addressing lysosomal dysfunction in AD and related conditions.^55^, ^84^ Despite known factors like presenilin 1^85^, ^86^ and Tyr682‐phosphorylated APP βCTF,^87^ the mechanisms underlying the weakening of V‐ATPase‐mediated lysosomal acidification remain unclear. This study highlights that ACLY regulates lysosomal acidification through the assembly of V‐ATPase subunits in AD model mice and N2a‐APP695 cells. Mechanistically, tubulin acetylation is essential for ACLY's role in V‐ATPase assembly, aligning with reports that increased acetylation of α‐tubulin promotes the assembly of V‐ATPase subunit domains.^60^, ^88^ Intriguingly, we found that ACLY also modulates the level of LC3‐II (Figures 4, 6), although the precise mechanism requires future investigation. Collectively, these results suggest that ACLY impacts both the formation of autophagosomes and downstream lysosomal function. This finding appears to contrast with a recent report by Frédéric Saudou et al.^89^ We posit that this discrepancy may stem from fundamental differences in the experimental models used and the methods of ACLY suppression. Together, these observations highlight that the regulatory role of ACLY in autophagy is highly context dependent.

Our study highlights the essential role of neuronal ACLY in cognition through its influence on tubulin acetylation and microtubule stability. It also demonstrates that ACLY deficiency exacerbates Aβ deposition by impairing V‐ATPase assembly and lysosomal acidification. Several important questions remain to be addressed in the future. First, the microtubule‐associated protein tau (MAPT) is a key regulator of microtubules in neurons and undergoes various post‐translational modifications, including phosphorylation, acetylation, and ubiquitylation. Recent studies have emphasized the role of tau acetylation in its pathological accumulation.^90^ ^−^ ^92^ Although ACLY has been shown to provide a substrate for tau acetylation,^93^ the impact of ACLY deficiency on tau pathology in the context of Aβ accumulation remains to be elucidated. Additionally, ACLY itself undergoes several post‐translational modifications, including phosphorylation, acetylation, and ubiquitination. While phosphorylation and acetylation enhance ACLY's enzymatic activity,^94^, ^95^ ubiquitination promotes its degradation.^96^ ^−^ ^98^ Investigating the mechanisms that affect the stability of neuronal ACLY protein could reveal new therapeutic strategies for AD. It is important to note that this study was conducted exclusively in male mice. Given the known sex differences in AD susceptibility and progression, future studies will be essential to determine whether our findings extend to females.

## AUTHOR CONTRIBUTIONS

Anlan Lin and Tianqing Han completed virus injection and performed the biochemical experiments and histopathological staining. Anlan Lin performed data analysis and wrote the first draft of the manuscript. Jianmin Chen performed all cell experiments. Xiaoman Dai completed virus injection and animal behavior analysis. Minxia Wu performed sample preparation and reading under electron microscope. Qiang Du performed partial data analysis. Jinbo Cheng completed the IHC analysis of AD brain samples. Wanjin Chen and Qinyong Ye participated the discussion of all data. Xiaochun Chen and Jing Zhang designed the study, wrote the paper, and supervised all aspects. All authors read and approved the final manuscript.

## CONFLICT OF INTEREST STATEMENT

The authors declare no competing interests. Author disclosures are available in the supporting information.

## CONSENT STATEMENT

*Post mortem* brain tissue was sourced from the National Human Brain Bank for Development and Function under irreversible anonymization. The ethics board of the Institute of Basic Medical Sciences, CAMS & PUMC waived re‐consent (Approval No. 009‐2014) based on pre‐existing donor agreements and complete de‐identification.

## Supporting information



Supporting information

Supporting information
