# Correction to “ACLY regulates autolysosome acidification through tubulin acetylation‐mediated assembly of V‐ATPase subunits in Alzheimer's disease model mice”

**DOI:** 10.1002/alz.71018

**Published:** 2025-12-13

Lin A, Dai X, Chen J, et al. ACLY regulates autolysosome acidification through tubulin acetylation‐mediated assembly of V‐ATPase subunits in Alzheimer's disease model mice. *Alzheimers Dement*. 2025;21(11):e70919. https://doi.org/10.1002/alz.70919.

1. In the Methods section 2.2, the original text from “The guide cannula was slowly lowered…” to “…collected for subsequent biochemical analysis.” was duplicated. This occurred because a revised, more objective description of the surgical procedure was inserted, but the original paragraph was inadvertently retained.

Correction: The original, duplicated paragraph has been deleted. The section now correctly contains only the revised text beginning with “The cannula was inserted into the prepared hole…”.

2. In the legend for Figure 7, the phrase “β‐actin was used as loading control” was redundant and unrelated to the core explanatory text of the legend.

Correction: This phrase has been removed to streamline the figure legend, as the loading control is already visually evident in the figure itself.

3. In Figure 2, the panel label “E” was incorrectly formatted, lacking parentheses.

Correction: This has been corrected to “(E)” to maintain consistent formatting with all other panel labels in the manuscript.

4. In Figure 4, the second instance of the panel label “(B)” was incorrect, resulting in a duplication.

Correction: This duplicate label has been corrected to “(D)” to reflect the proper sequence and ensure accurate figure navigation.

We apologize for these errors and any inconvenience they may have caused.

