# Supplementary material for: ACLY regulates autolysosome acidification through tubulin acetylation‐mediated assembly of V‐ATPase subunits in Alzheimer's disease model mice
Source: Alzheimers Dement. 2025 Nov 19;21(11):e70919. doi: 10.1002/alz.70919 (PMC12627967; doi:10.1002/alz.70919)
Supplement: Supplementary file 1 — Supporting information [file ALZ-21-e70919-s002.pdf]

Supplementary Materials

Table S1. Information About Human Samples

Case	PMI (h)	Age (years)	Sex	Disease stage
Control 1	21.5	78	Male	Braak 0
Control 2	4.3	70	Male	Braak 0
Control 3	23.5	93	Male	Braak 0
Control 4	4	95	Male	Braak 0
Control 5	3	89	Male	Braak 0
AD 1	6	93	Male	Braak I
AD 2	9.5	94	Male	Braak I
AD 3	8.8	87	Male	Braak I
AD 4	13.5	78	Male	Braak I
AD 5	4	77	Male	Braak I

Postmortem interval (PMI) is presented in hours between time of patient death and necropsy.

Table S2. Key source table

REAGENT or RESOURCE	SOURCE	IDENTIFIER
**Antibodies**		
ACLY	Proteintech<br>Abcam	15421-1-AP<br>ab40793
NeuN	Abcam	ab177487
GFAP	SYSY	173011
Iba1	Abcam	ab5076
APP	Millipore	17160
α-tubulin	Proteintech	66031-1-Ig
β-III-tubulin	Abcam	ab78078
AceCS1	CST	3658S
β-actin	Abcam	ab8226
p62	CST	5114<br>23212
ATP6V1A	Santa Cruz	sc-293336
ATP6V1C1	Santa Cruz	sc-271077
ATP6V0D	Santa Cruz	sc-393322
Acetyl-α-tubulin	Sigma	T7451
Acetyl-H3	Abcam	ab47915
Cathepsin B	CST	31718
LAMP1	Abcam	ab25245

Mouse monoclonal anti-6E10 Antibody	Biolegend	803001
Synaptophysin	CST	9020S
PSD95	Abcam<br>Proteintech	ab18258<br>20665-1-AP
H3	Proteintech	17168-1-AP
PNFH	Biolegend	835503
Acetyl-lysine	Invitrogen	MA1-2021
Map2	Proteintech	17490-1
NaKATPase	Abcam	Ab76020
LAMP2A	Abcam	Ab18528
CathepsinD	Abcam	AB75852
LC3B	Abcam	Ab192890
HRP-conjugated goat anti-rabbit IgG	Abcam	ab205718
HRP-conjugated goat anti-mouse IgG	Abcam	ab205719
Alexa Fluor 488-conjugated donkey anti-rabbit IgG	Invitrogen	A21206
Alexa Fluor 594-conjugated<br>donkey anti-mouse IgG	Invitrogen	A21203
Alexa Fluor 647-conjugated<br>donkey anti-mouse IgG	Invitrogen	A32795
Alexa Fluor 405-conjugated<br>goat anti-mouse IgG	Abcam	ab175560
**Biological samples**		
Paraffin-embedded postmortem brain tissues from patients with AD and healthy controls	National Human Brain Bank for Development and Function at the Chinese Academy of Medical Sciences and Peking Union Medical College in Beijing, China.	N/A
**Chemicals**		
Nicotinamide	Sigma-Aldrich	V900517
Trichostatin A	Sigma-Aldrich	T1952
Thioflavine S	Merck	T1982-25G

Methoxy-X04	MCE	HY-103240
**Critical commercial assays**		
Scientific Cell Membrane Protein Extraction and Isolation Kit	Thermo	YD371740
MCE Cell Counting Kit-8	MCE	HY-K0301-100T
Human Aβ42 ELISA Kit	Invitrogen	KHB3441
Mouse Ac-CoA ELISA Kit	Shanghai Yiyan Biotech Co. Ltd	EY12009-M
Cathepsin B activity assay kit	Abcam	AB65300
LysoSensor™ Yellow/Blue DND-160	Thermo Fisher	L7545
**Critical experimental drugs**		
ACLY Inhibitor-BMS	MCE	BMS-303141
**Viral vector**		
pAAV-hSyn-MCS-3×FLAG-tWPA (control)	HeYuan Biological Company (Shanghai, China).	N/A
pAAV-hSyn-ACLY-3×FLAG-tWPA (5×FAD-oeACLY)		
pAAV-hSyn-EGFP-3×flag miR30shRNA(scrambled)-WPRE (control)		N/A
pAAV-hSyn-EGFP-3×flag-miR30shRNA (ACLY)-WPRE (5×FAD-shACLY)		
**Experimental models: Organisms/strains**		
Mouse: 5×FAD	Inhouse breeding	N/A
Mouse: APP/PS1	Inhouse breeding	N/A
Mouse: C57BL/6J		N/A
**Oligonucleotides**		
Primers for RT-qPCR, see Table S3	This paper	N/A
**Software and algorithms**		
Etho-vision video tracking software	Noldus	N/A
GraphPad Prism 9.0	GraphPad software	https://www.graphpad.com/
ImageJ software	National Institutes of Health	https://imagej.nih.gov/ij/downloa
**Other**		
Guide cannula	RWD	Cat# 62070

Stainless-steel cap	RWD	Cat# 62170

Table S3. List of primers used in this study

Gene	Forward primer sequence (5'→3')	Reverse primer sequence (5'→3')
ACLY	CGCACCATAGCCATCATAGC	TGTCCAGCATTCCACCAGTA
NAT3	AGTCCATGGCTTAATGGGCA	GCACAAGCTTTGTCTCCAAGT
HDAC6	TGCTCTAGTTCTAGGCTTGTCAT	AGGTAGTGCTGCTATGGTCTT

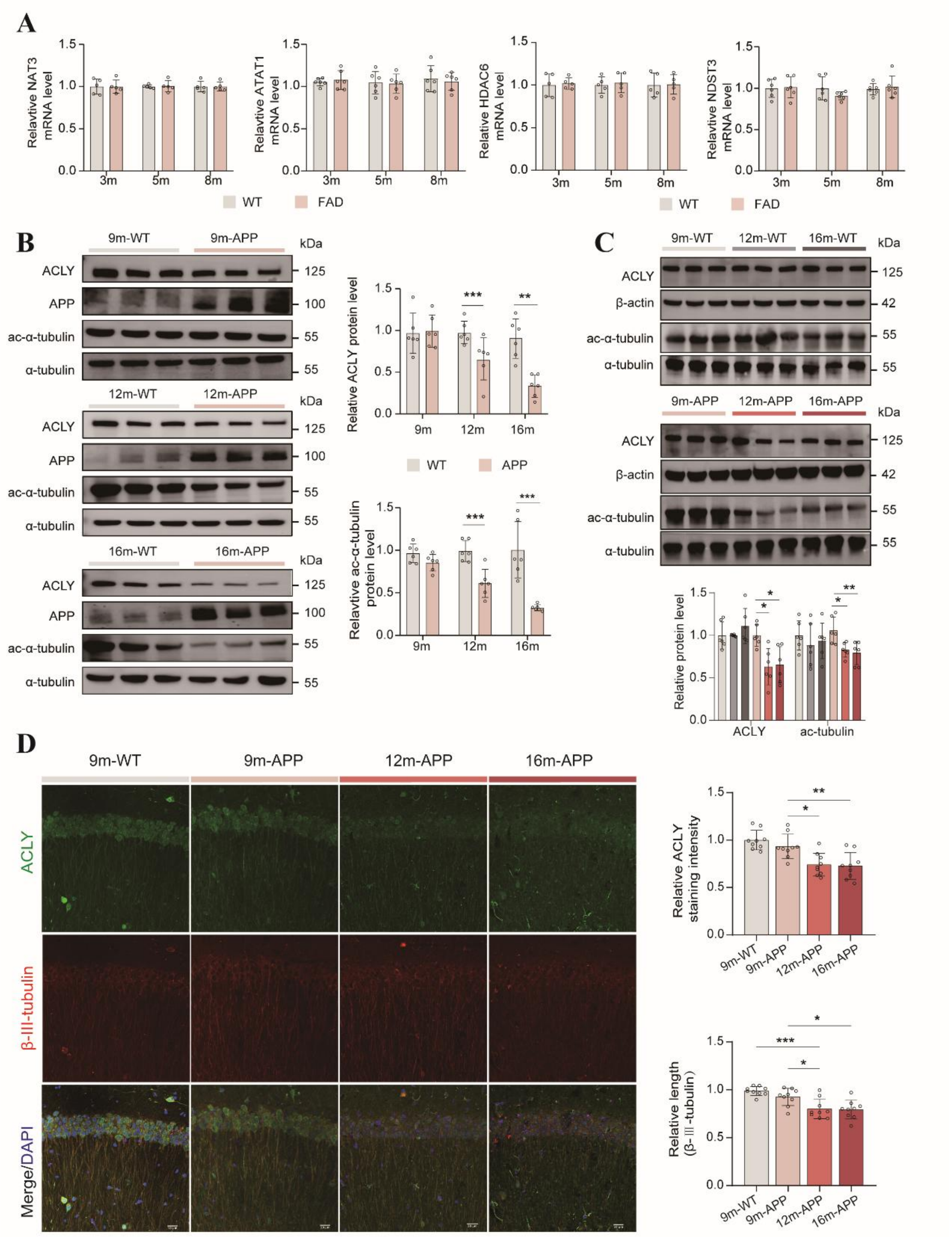

Figure S1. Decreased the level of neuronal ACLY and ac-α-tubulin in APP/PS1 mice, related to Figure 1.

(A) Quantitative analyses of the mRNA level of acetyltransferase NAT3, ATAT1 and deacetylase HDAC6, NDST3 for tubulin in the dHip. n = 6 mice per group.

(B-C) Representative immunoblots and quantitative analyses of ACLY and ac-α-tubulin

in the dorsal hippocampus (dHip). n =5-6 mice per group.

(D) IF staining of ACLY (green) and β-III-Tubulin (red) in the hippocampal CA1 region (left), quantifications of ACLY and β-III-Tubulin (right). Scale bar=100 μm. n = 9 slices from 3 mice per group.

Data were expressed as mean ± SEM. $^{*}p < 0.05$, $^{**}p < 0.01$, $^{***}p < 0.001$. Two-tailed unpaired Student's t test was used in A and B. One-way ANOVA with Tukey's multiple comparisons test was used in C and D.

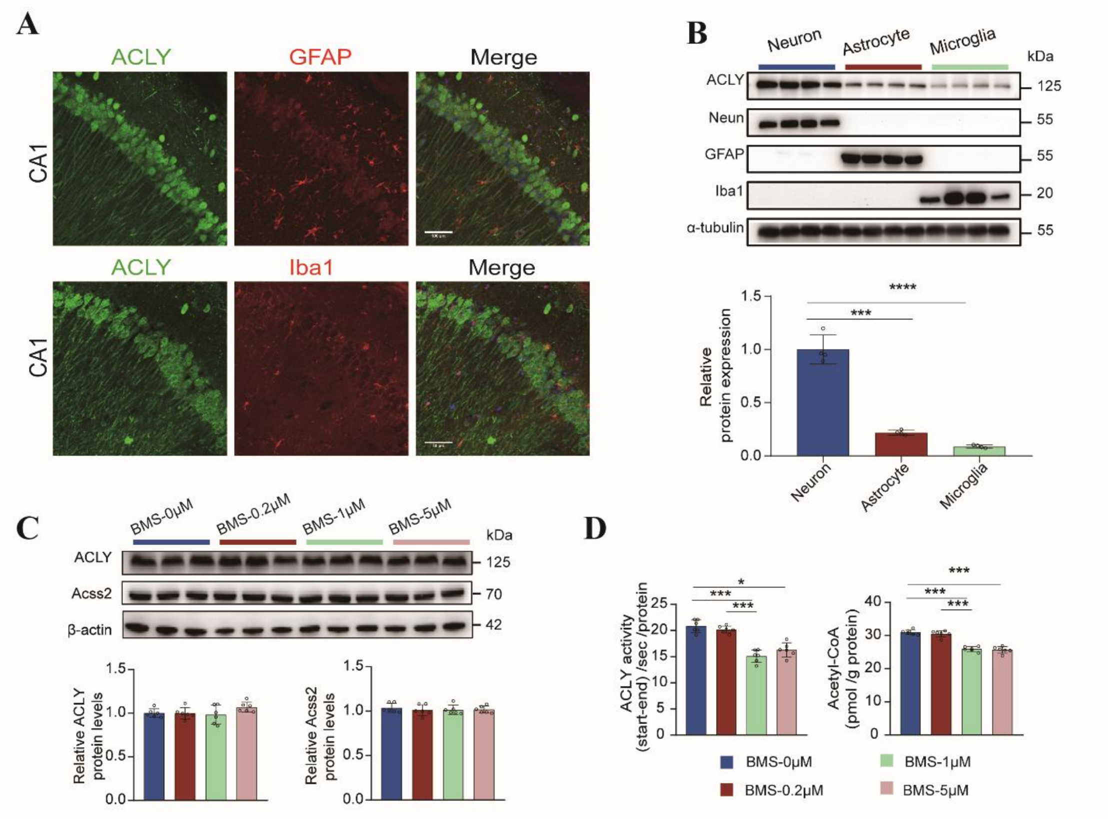

Figure S2. The physiological functions of ACLY, related to Figure 2.

(A) IF staining showed the distribution of ACLY, GFAP, Iba1 in the dHIP. Scale bar=100 μm, n = 3 mice.

(B) Western blotting analysis for the expression of ACLY in primary culture cells. NeuN, GFAP and Iba1 were used as marker genes in neurons, astrocytes and microglia, respectively. n = 4.

(C) Representative immunoblots and quantitative analyses of ACLY and ACSS2 in the

primary neuron, n = 6.

(D) Detection of ACLY enzymatic activity (left) and quantification of acetyl-CoA level (right) in the primary neuron, n = 6.

Data are expressed as mean ± SEM. $^{*}p < 0.05$, $^{**}p < 0.01$, $^{***}p < 0.001$, $^{****}p < 0.0001$; One-way ANOVA with Tukey's multiple comparisons test was used.

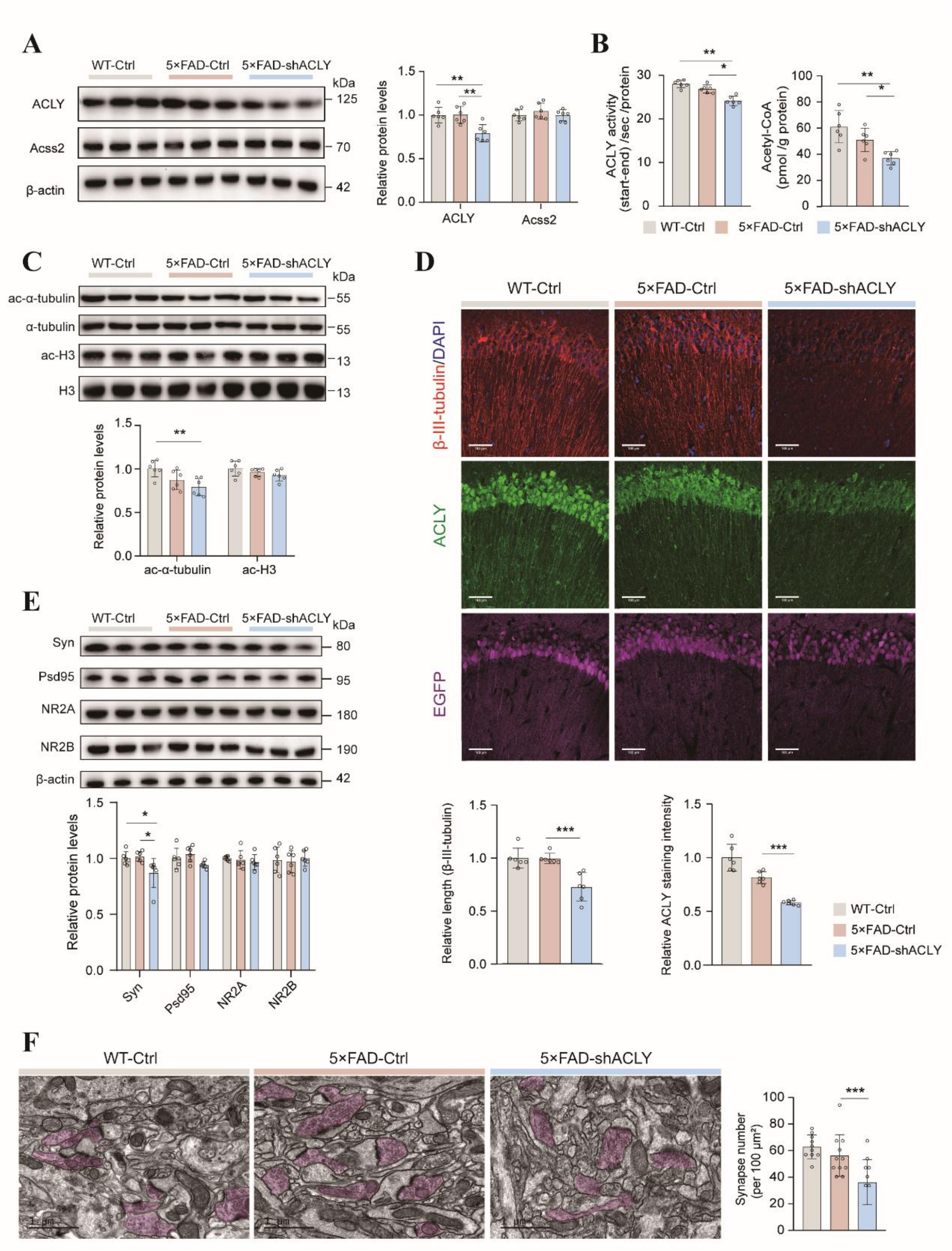

Figure S3. Knockdown of neuronal ACLY impaired microtubule stability and synaptic plasticity in the early stage of 5×FAD mice, related to Figure 4.

(A, C, E) Representative immunoblots and quantitative analyses of dorsal hippocampus lysates for indicated proteins. n = 6 mice per group.

(B) Detection of ACLY enzymatic activity (left) and quantification of acetyl-CoA level (right) in the dHip. n = 6 mice per group.

(D) Representative images (up) and quantification (down) of hippocampus immunostained with the ACLY and β-III-tubulin antibody. Scale bars=100 µm. n = 6 slices from 3 mice per group.

(F) Representative TEM images and quantitative analyses of the typical synaptic structure of hippocampus CA1. Red areas indicate for presynaptic regions. Scale bar=1 µm. n = 3 mice pre group.

Data are expressed as mean ± SEM. $^{*}p<0.05$, $^{**}p<0.01$. One-way ANOVA with Tukey's multiple comparisons test was used.

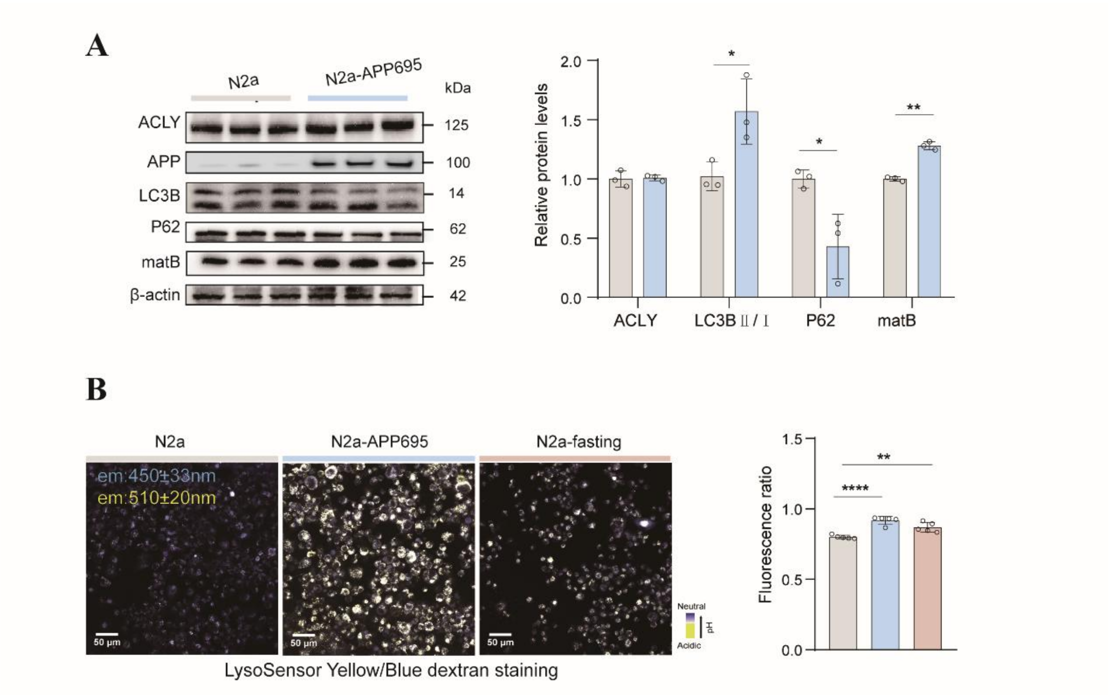

Figure S4. Increased autophagic lysosomal flux and lysosomal acidification in N2A-APP695 cells, related to Figure 7.

(A) Representative immunoblots and quantitative analysis of cells lysates for indicated proteins. β-actin was used as loading control. Normalized by N2a-Ctrl group. n =3 per group.

(B) Determination of lysosomal pH with the ratiometric probeLysoSensor Yellow/Blue dextran. Fasting for 12h of N2A cells was used as a positive control.

Data are expressed as mean ± SEM. $^{*}p < 0.05$, $^{**}p <0.01$, $^{****}p <0.0001$, The unpaired two-tailed t-test were used in A, One-way ANOVA with Tukey's multiple comparisons

was used in B. Data are representative of 2 independent experiments.

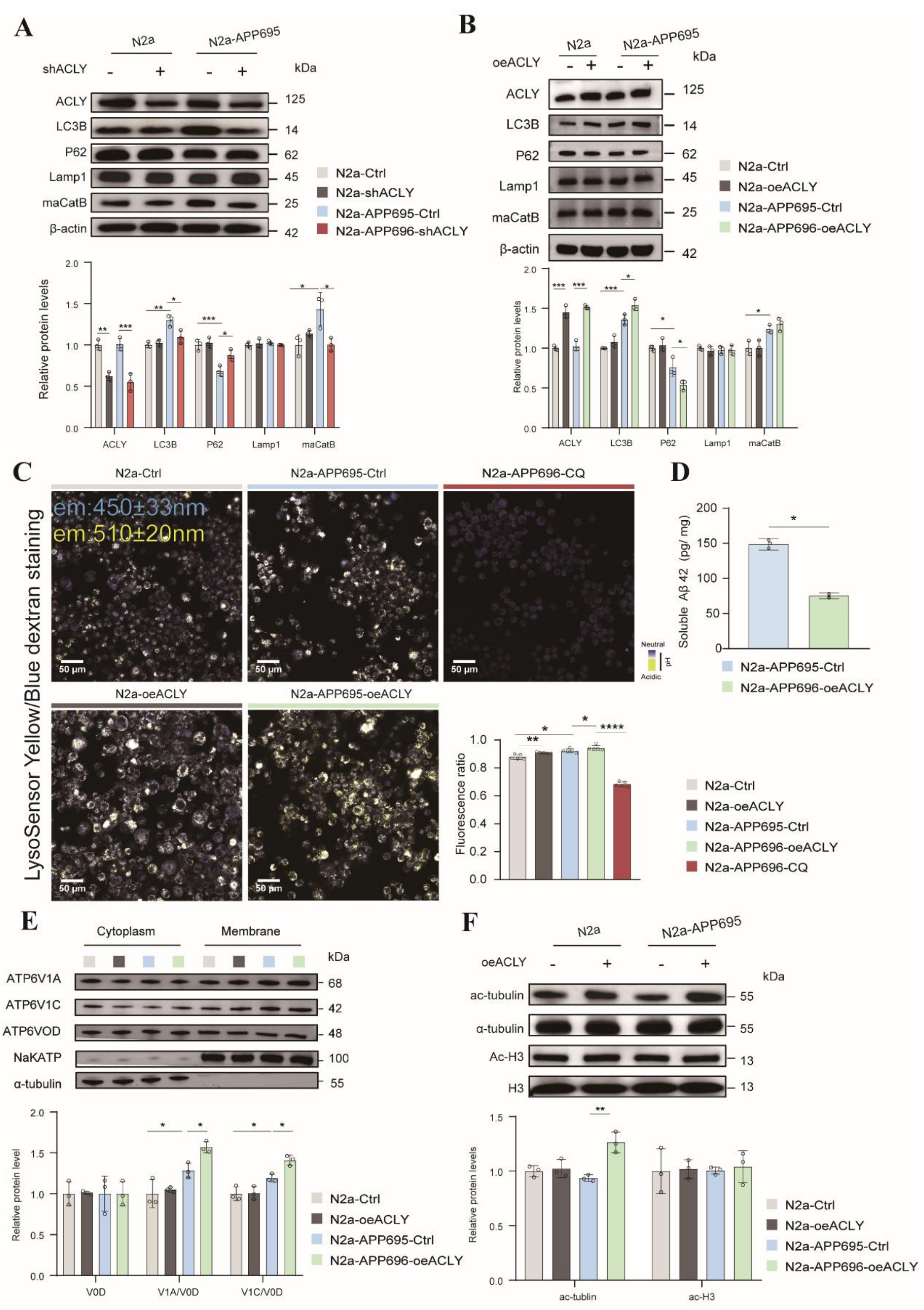

Figure S5. Upregulation of ACLY expression increases lysosomal function through enhancing the assembly of vATPase mediated by ac-α-tubulin, related to Figure 7.

(A, B) Representative immunoblots and quantitative analysis of cells lysates for indicated proteins. β-actin was used as loading control. Normalized by N2a-Ctrl group. n = 3 per group.

(C) Determination of lysosomal pH with the ratio metric probe LysoSensor Yellow/Blue dextran. Cell lines treated with 50 μM Chloroquine (CQ) for 12 h was used as a positive control. Scale bar =50 μm. n = 5 per group. Repeat the experiment for 3 times.

(D) ELISA analysis the level of soluble Aβ42 in cell lysates. n = 3 per group.

(E, F) Representative immunoblots and quantitative analyses of the vATPase V1-V0 holoenzyme in cells (E) and cells lysates for indicated proteins (F). Na-K-ATPase and α-tubulin are used as markers for membrane proteins and cytoplasm protein, respectively. n = 3 per group.

Data are expressed as mean ± SEM. $^{*}p < 0.05$, $^{**}p < 0.01$, $^{***}p < 0.001$, $^{****}p < 0.0001$, Two-way ANOVA with Tukey's multiple comparisons was used except for Fig. D. Data are representative of 2 independent experiments.
